# Alpelisib and Fulvestrant in PIK3CA-mutated hormone receptor-positive HER2-negative advanced breast cancer included in the German PRAEGNANT trial

**DOI:** 10.1007/s10549-026-07939-z

**Published:** 2026-04-02

**Authors:** Manuel Hörner, Lara M. Tretschock, Nelson John, Philipp Ziegler, Lothar Häberle, Sabrina Uhrig, Chloë Goossens, Niklas Amann, Jan-Philipp Cieslik, Dominik Dannehl, Thomas M. Deutsch, Moritz Dimpfl, Max Ehlert, Kathleen Eichstädt, Alexander Englisch, Melitta B. Köpke, Annika Krückel, Theresa Link, Annika Müller, Kristin Reinhardt, Jonas Roth, Henning Schäffler, Lea Sych, Christian M. Tegeler, Catharina Wichmann, Maggie Banys-Paluchowski, Henriette Princk, Achim Rody, Sara Y. Brucker, Nina Ditsch, Johannes Ettl, Tanja Fehm, Carolin C. Hack, Peyman Hadji, Alexander Hein, Wolfgang W. Janni, Hans-Christian Kolberg, Diana Lüftner, Michael P. Lux, Volkmar Müller, Andreas Schneeweiss, Florin-Andrei Taran, Hans Tesch, Diethelm Wallwiener, Frederik Marmé, Stephan Seitz, Erik Belleville, Andreas Hartkopf, Laura L. Michel, Markus Wallwiener, Peter A. Fasching, Nikolas Tauber

**Affiliations:** 1 Department of Gynecology and Obstetrics, University Hospital Erlangen, Comprehensive Cancer Center Erlangen-EMN (CCC ER-EMN), Friedrich-Alexander-Universität Erlangen-Nürnberg (FAU), Erlangen, Germany; 2 Department of Obstetrics and Gynecology, University Hospital Heidelberg, Heidelberg, Germany; 3 Biostatistics Unit, Department of Gynecology and Obstetrics, Erlangen University Hospital, University Hospital Erlangen, Friedrich-Alexander-Universität Erlangen-Nürnberg (FAU), Comprehensive Cancer Center Erlangen-EMN (CCC ER-EMN), Erlangen, Germany; 4 Department of Gynecology and Obstetrics, Center of Integrated Oncology ABCD, Medical Faculty, Heinrich Heine University and University Hospital Düsseldorf, Moorenstrasse 5, Düsseldorf, Germany; 5 Department of Gynecology and Obstetrics, University Hospital Tübingen, Tuebingen, Germany; 6 Department of Gynecology and Obstetrics, Medical Faculty Mannheim, University Medical Center Mannheim, Heidelberg University, Mannheim, Germany; 7 University Medical Center Hamburg-Eppendorf, Hamburg, Germany; 8 Department of Gynecology, University Hospital Halle, Halle, Germany; 9 Department of Obstetrics and Gynaecology, University Hospital Augsburg, Augsburg, Germany; 10 Department of Gynecology and Obstetrics, Medical Faculty and University Hospital Carl Gustav Carus, Technische Universität Dresden, Dresden, Germany; 11 Department of Obstetrics and Gynecology, University Medical Center Regensburg, Caritas Hospital St. Josef, University of Regensburg, Regensburg, Germany; 12 Department of Gynecology and Obstetrics, University Hospital Ulm, Ulm, Germany; 13 Department of Gynecology and Obstetrics, University Hospital Schleswig-Holstein, Campus Lübeck, Luebeck, Germany; 14 Department of Obstetrics and Gynecology, Klinikum Rechts Der Isar, Technical University of Munich, Munich, Germany; 15 Cancer Center Kempten/Allgäu (CCKA), Klinikum Kempten, Kempten, Germany; 16 Frankfurt Center for Bone Health and Endocrinology, Frankfurt Am Main, Germany; 17 Department of Gynecology, Klinikum Esslingen GmbH, Esslingen, Germany; 18 Department of Gynecology and Obstetrics, Marienhospital Bottrop, Bottrop, Germany; 19 Immanuel Hospital Märkische Schweiz & Immanuel Campus Rüdersdorf, Medical University of Brandenburg Theodor-Fontane, Rüdersdorf Bei Berlin, Germany; 20 Department of Gynecology and Obstetrics, Frauenklinik St. LouiseSt. Josefs-KrankenhausVincenz Kliniken GmbH, PaderbornSalzkottenPaderborn, Germany; 21 National Center for Tumor Diseases, University Hospital and German Cancer Research Center Heidelberg, Heidelberg, Germany; 22 German Cancer Research Center (DKFZ), Heidelberg, Germany; 23 Department of Gynecology and Gynecologic Oncology, Center for Integrated Oncology Aachen Bonn Cologne Düsseldorf, Medical Faculty and University Clinic of Cologne, University of Cologne, Cologne, Germany; 24 Oncology Practice, Bethanien Hospital, Frankfurt Am Main, Germany; 25 ClinSol GmbH & Co KG, Würzburg, Germany

**Keywords:** Advanced breast cancer, Real-world data, Oral cancer agents

## Abstract

**Purpose:**

Mutations in *PIK3CA* are one of several actionable mutations for patients with hormone receptor positive, human epidermal growth factor receptor 2 negative breast cancer. Alpelisib in combination with fulvestrant was the first approved PI3K inhibitor and was introduced in clinical practice in 2019. A lack of evidence for the use of alpelisib in the context of current treatment options like cyclin-dependent 4/6 inhibitor (CDK4/6i), highlights the importance of this analysis. We provide a real-world analysis of the use of alpelisib with the prospective German PRAEGNANT registry (NCT02338167).

**Methods:**

57 patients with advanced breast cancer receiving alpelisib and fulvestrant were identified. 55 Patients had received prior CDK4/6i therapy. Progression-free survival (PFS) and overall survival (OS) were calculated for all patients, and stratified according CDK4/6i pre-treatment, using the Kaplan–Meier method. Subgroups (age, line of therapy, concomitant disease among others), somatic *PIK3CA* mutations, reasons for discontinuation and adverse events (AEs) were analyzed.

**Results:**

The median PFS was 5.0 (95% confidence interval [CI], 3.1–9.4) months, and the median OS was 20.1 (95% CI, 14.6–30.8) months. Line of therapy and concomitant diseases appeared to affect PFS, while the line of therapy and preexisting diabetes influenced OS. However, subgroups were too small for statistical testing. Discontinuation was mainly due to tumor progression (56.1%). Hyperglycemia, rash and diarrhea were the most documented AEs.

**Conclusion:**

This prospective real-world analysis shows slightly shorter median PFS and OS times compared with the pivotal trials. Patients in our analyses received alpelisib in later therapy lines, which may explain the poorer outcome.

**Supplementary Information:**

The online version contains supplementary material available at 10.1007/s10549-026-07939-z.

## Introduction

In advanced breast cancer (ABC), hormone receptor positive (HRpos) and human epidermal growth factor receptor 2 negative (HER2neg) represent the most common subtype at around 70% [1]. In this group, approximately 40% of patients harbor cancer cells with a somatic mutation in the Phosphatidylinositol-4,5-bisphosphate 3-kinase Catalytic Subunit Alpha (*PIK3CA)* gene, which encodes the p110α-subunit of class IA phosphatidylinositol 3-kinase (PI3K) [2, 3]. While *PIK3CA* mutated tumors are described to have poor outcomes, PIK3CA mutations represent a potential target for personalized treatments [4, 5]. Three approved drugs already target the *PIK3CA*/*AKT1*/*PTEN* pathway(alpelisib, capivasertib, and inavolisib), while other actionable targets include *ESR1, HER2*and *PD-(L)1*, making individualized treatment a current reality in the treatment of ABC [6–11].

Alpelisib is the first approved drug to target the *PIK3CA*/*AKT1*/*PTEN* pathway. It is an orally available α-selective PI3K inhibitor and degrader that was investigated in the Phase III SOLAR-1 trial [12]. In SOLAR-1, alpelisib/fulvestrant was tested against fulvestrant/placebo and showed prolonged progression-free survival (PFS), with 11.0 (95% confidence interval [CI], 7.5–14.5) versus 5.7 (95% CI, 3.7–7.4) months. This led to its Food and Drug Administration approval in May 2019 [8].

In SOLAR-1, overall survival (OS) results were numerically but not statistically significantly different between the two treatment groups, and only 5.9% of patients had received a prior cyclin-dependent 4/6 inhibitor (CDK4/6i). The Phase II BYLieve trial subsequently reported PFS data following prior CDK4/6i therapy. The PFS in cohort A, with patients pre-treated with a CDK4/6i plus letrozole receiving alpelisib plus fulvestrant (127 patients), was reported to be 8.0 (95% CI, 5.6–8.6) months [13]. For cohort B, alpelisib plus letrozole after CDK4/6i plus fulvestrant also showed activity, with a reported PFS of 5.7 months [14]. Most patients in both cohorts received alpelisib plus endocrine therapy in the second line.

Real-world data (RWD) from an Early Access Program (EAP) in France reported PFS to be 5.3 (95% CI, 4.7–6.0) months in a heavily pre-treated cohort with 233 patients [15]. The median number of prior treatment lines was 4. 227 (97.4%) had received prior CDK4/6i therapy, 180 patients (77.3%) had received prior chemotherapy, 175 (75.1%) prior fulvestrant alone or in combination and 131 (56.2%) had received prior everolimus plus endocrine treatment.

As alpelisib is included in international guidelines and algorithms following progression on CDK4/6i therapy and is currently available in many countries, the objectives of this analysis were to determine progression free survival and the median overall survival (PFS and OS) for all patients in the PRAEGNANT registry who received alpelisib, and to identify potential factors influencing PFS and OS in these patients.

## Methods

### The PRAEGNANT research network

The PRAEGNANT (Prospective Academic Translational Research Network for the Optimization of the Oncological Health Care Quality in the Adjuvant and Advanced/Metastatic Setting, NCT02338167, [16]) study is an ongoing, prospective breast cancer registry. Documentation is similar to that of a clinical trial; the first patient was recruited in July 2014. The PRAEGNANT study aims to assess treatment patterns, to investigate quality of life and survivorship, to answer translational research questions and finally to identify patients’ eligibility for clinical trials or specific targeted treatments [16–19]. Patients can be included at any point during the course of their disease. Follow-up assessments for the advanced setting are updated every 3 months until month 24 and thereafter every 6 months in case there is no progression or change of therapy within 3 months of observation. Furthermore, biomaterials from blood and tumor biopsies are collected for research purposes [16]. The study was approved by the relevant ethics committees. All patients included in the present study provided informed consent.

### Patients

**Fig. 1:**
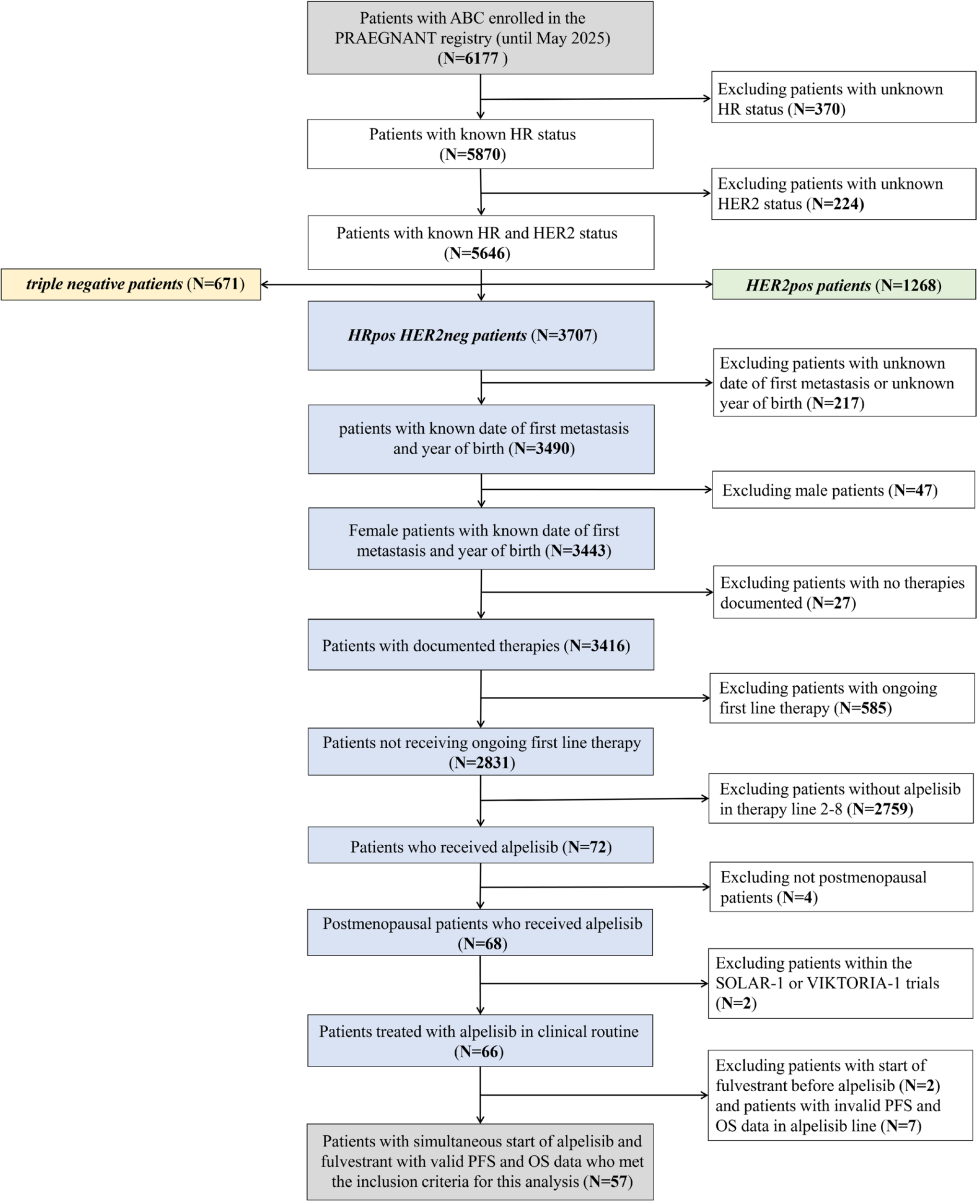
Patient flow chart showing exclusion reasons and the final patient population

At the time of data cut-off (April 14, 2025), 6,177 ABC patients were registered in the PRAEGNANT registry. Of these, 5,646 patients had a documented hormone receptor (HR) and human epidermal growth factor receptor 2 (HER2) status. Of this overall population, 1268 patients had HER2-positive disease, 671 patients had triple-negative disease and 3,707 patients had HRpos, HER2neg ABC. From this population relevant to our analysis, 217 patients had to be excluded due to missing documentation of the date of first metastasis or unknown year of birth. Male patients were excluded because of the very small sample size (*N* = 1 remaining after other exclusion criteria), to maintain statistical homogeneity, and in line with the usual PRAEGNANT analysis practice. Premenopausal patients were excluded in accordance with the product label for legal reasons, as their inclusion would have constituted off-label use. A total of 585 patients without disease progression on first line endocrine based therapy were excluded, resulting in 2831 female patients with HRpos, HER2neg ABC who were eligible for treatment with alpelisib. 2759 patients not receiving alpelisib in lines 2–8 were excluded. Of the remaining 72 HRpos HER2neg patients who did receive alpelisib, four were not postmenopausal, two received alpelisib within the SOLAR-1 or the VIKTORIA-1 trial, two started fulvestrant prior to alpelisib in the same therapy line, and seven patients had invalid PFS or OS follow-up data. Consequently, 57 patients were included in the final patient population. The patient flow chart is shown in Fig. 1. Of note, 917 patients from the study population had died before alpelisib was approved.

### Data ascertainment

Data was collected by trained personnel and documented in an electronic case report form [16]. Automated plausibility checks and on-site monitoring were performed. Data usually not recorded in everyday clinical work was prospectively collected using structured questionnaires completed on paper (epidemiological data such as family history, cancer risk factors, quality of life, nutrition and lifestyle items, and psychological health).

### Definition of grading, HR and HER2 status

The definition of HR status, HER2 status, and grading has been described previously [17]. If a biomarker assessment of the metastatic site was available, this receptor status was used for the analysis. If there was no information for metastases, the latest biomarker results from the primary tumor were used. Additionally, all patients who received endocrine therapy in the metastatic setting were assumed to be HRpos, and all patients who had ever received anti-HER2 therapy were considered to be HER2-positive. There was no central review of biomarkers. The study protocol recommended assessing estrogen receptor and progesterone receptor status as positive if ≥ 1% was stained. A positive HER2 status was defined by an immunohistochemistry score of 3 + or positive fluorescence in situ hybridization/chromogenic in situ hybridization. Patients receiving alpelisib were assumed to be *PIK3CA*-mutated.

### Statistical Analysis

Patient and tumor characteristics were described using appropriate summary statistics. Mean and standard deviation were calculated for continuous variables, while frequency and percentage were used for categorical variables.

PFS was defined as the time from the start of therapy to the earliest occurrence of disease progression (distant-metastasis, local recurrence, or death from any cause), or the last known date the patient was progression-free. The analysis was left-truncated at the time of study entry if entry occurred after the initiation of therapy. OS was defined similarly.

The primary objective was to investigate the PFS and OS rates and times in patients receiving alpelisib. Survival rates with 95% CIs and median survival times were estimated using the Kaplan–Meier product-limit method. Subgroup analyses were conducted based on the following variables: age (categorical; until 49 years, 50–74 years, 75 + years), body mass index (BMI) (categorical; normal, overweight/obese), Eastern Cooperative Oncology Group (ECOG) score (ordinal; 0–2), grading (ordinal; G1, G2, G3), line of therapy (ordinal; 2–8), metastasis timing (categorical; de novo metastasis, metastasis ≤ 60 months after primary diagnosis, metastasis > 60 months after primary diagnosis), concomitant diseases (ordinal; 0–5) and diabetes (categorical; yes, no). The 95% CI for the median survival time was calculated using the Brookmeyer and Crowley method [20]. The same approach was applied for OS. Cox proportional hazards regression was not performed due to the limited sample size.

Survival analyses were also carried out for the subgroup of patients who had received prior CDK4/6i therapy as first-line treatment followed by second line alpelisib. Hereby, patients were stratified according to the duration of first-line CDK4/6i therapy (< 24 months vs ≥ 24 months, as this roughly corresponds to the median PFS of first line CDK4/6i therapy in the relevant trials [21–24]). Further survival analyses were performed for patient groups according to the PIK3CA mutation (p.E542K, p.H1047R, p.E545K, other). Six patients with double mutations were classified, according to current literature, into the more aggressive mutation group (Table S4, supplementary section) [25, 26].

Calculations were performed using the R system for statistical computing (version 4.3.0; R Development Core Team, Vienna, Austria, 2023).

## Results

### Patient characteristics

Analyses were made on the final population for patients receiving alpelisib (N = 57) as described above. Demographic and baseline tumor characteristics are shown in Table 1. The mean age was 64.2 years. Most patients (55 of 57) had received prior CDK4/6i. 36.8% (N = 21) of patients received alpelisib in the second line of therapy for ABC, with a relatively even distribution thereafter up to the eighth line of therapy. Visceral metastasis was observed in 61.4% (N = 35) of patients, 15.8% (N = 9) had bone-only metastasis, and 5.3% (N = 3) had brain metastasis. 29.4% (N = 15) of patients had a de novo metastatic disease, 23.5% (N = 12) developed metastatic disease ≤ 60 months, and 47.1% > 60 months after primary diagnosis. Concomitant diseases at the start of alpelisib treatment were present in 82.5% of the patients, with 29.8% having three or more. Mean BMI was 24.6 kg/m^2^, and eight patients (15.1%) presented with diabetes (type 1 or type 2) before treatment indication with alpelisib. Specification of observed *PIK3CA* mutations is listed in the supplements section (Table S4), with p.H1047R (N = 13, 22.8%), p.E454K (N = 10, 17.5%), and p.E542K (N = 9, 14.8%) being most often described. Follow-up therapies in the therapy line after alpelisib and fulvestrant are listed in the supplementary section (Table S5).

**Table 1:** Patient and tumor characteristics, showing mean and standard deviation (SD), median and interquartile range (IQR), or frequency and percentage

		All Patients (N = 57)
Age (years)	Mean (SD)	64.2 (9.5)
	Median (IQR)	64.0 (58.0, 70.0)
	Up to 49	3 (5.3)
	50 to 74	45 (78.9)
	75 +	9 (15.8)
BMI (kg/m2)	Mean (SD)	24.6 (4.1)
	Median (IQR)	23.7 (21.6, 27.0)
	< 18.5 (Underweight)	2 (3.6)
	18.5–24.9 (Normal)	32 (58.2)
	25.0–30.0 (Overweight)	14 (25.5)
	> 30.0 (Obese)	7 (12.7)
ECOG	0	37 (66.1)
	1	16 (28.6)
	2	3 (5.4)
Grading	1	2 (3.8)
	2	33 (63.5)
	3	17 (32.7)
Line of Therapy	2	21 (36.8)
	3	10 (17.5)
	4	10 (17.5)
	5	7 (12.3)
	6	6 (10.5)
	7	2 (3.5)
	8	1 (1.8)
Metastasis pattern	Brain	3 (5.3)
	Visceral	35 (61.4)
	Bone only	9 (15.8)
	Others	10 (17.5)
Metastasis timing	De novo metastasis	15 (29.4)
	Metastasis ≤ 60 months	12 (23.5)
	Metastasis > 60 month	24 (47.1)
Concomitant diseases	0	10 (17.5)
	1	14 (24.6)
	2	16 (28.1)
	3–5	13 (22.8)
	> 5	4 (7.0)
Diabetes	Yes	8 (15.1)
	No	45 (84.9)
Previous CDK4/6 therapies	0	2 (3.5)
	1	49 (86.0)
	2	6 (10.5)
Previous chemotherapies	0	31 (54.4)
	1	13 (22.8)
	2	7 (12.3)
	3	4 (7.0)
	4	2 (3.5)
Previous antihormone therapies	0	1 (1.8)
	1	26 (45.6)
	2	14 (24.6)
	3	12 (21.1)
	4	3 (5.3)
	5	1 (1.8)

### Patient disposition

Regarding PFS, the median observation time was 5.0 months (interquartile range [IQR]: 2.1–11.9 months); the number of events was 53. Regarding OS, the median observation time was 14.5 months (IQR: 5.9–29.6 months); the number of events was 40. While most patients (N = 32, 56.1%) discontinued treatment with alpelisib and fulvestrant due to tumor progression, three patients (5.3%) wished to discontinue the alpelisib therapy, three patients (5.3%) died during treatment, and three further patients stopped treatment for unknown reasons. Treatment was discontinued due to toxicity in 10 (17.5%) patients. At the time of analysis, six (10.5%) patients were still receiving treatment with alpelisib and fulvestrant.

### Progression-free survival

The median PFS was 5.0 months (95% CI, 3.1–9.4 months, Table 2). Six-month, 12-month, and 24-month PFS rates with 95% CI with regard to different cofactors (age, line of therapy, metastasis timing, concomitant diseases, diabetes pre-alpelisib, BMI, ECOG, tumor grading, metastasis sites, PIK3CA mutation and duration of first-line CDK4/6 therapy in patients receiving second line alpelisib) are presented in Table 2.

**Table 2:** Median progression-free survival times and survival rates

Outcome		N	Events	Median survival time (95% CI) in months	6-month survival rate (95% CI)	12-month survival rate (95% CI)	24-month survival rate (95% CI)
All patients		57	53	5.0 (3.1, 9.4)	0.46 (0.35, 0.61)	0.28 (0.19, 0.43)	0.14 (0.07, 0.27)
Age	Up to 49	3	3	5.0 (2.1, NA)	0.33 (0.07, 1.00)	0.33 (0.07, 1.00)	0.00^2^
	50—74	45	41	5.9 (3.8, 11.1)	0.50 (0.37, 0.67)	0.32 (0.20, 0.49)	0.18 (0.09, 0.35)
	75 +	9	9	2.7 (2.0, NA)	0.33 (0.13, 0.84)	0.11 (0.02, 0.71)	0.00^2^
BMI	Normal	32	31	5.8 (2.8, 9.5)	0.48 (0.34, 0.70)	0.26 (0.14, 0.47)	0.13 (0.05, 0.32)
	Overweight/Obese	21	18	5.9 (3.1, NA)	0.48 (0.30, 0.75)	0.33 (0.18, 0.61)	0.10 (0.02, 0.53)
ECOG	0	37	36	5.8 (3.1, 11.1)	0.49 (0.35, 0.68)	0.30 (0.18, 0.49)	0.15 (0.07, 0.33)
	1 or 2	19	16	5.3 (2.7, NA)	0.44 (0.27, 0.74)	0.28 (0.13, 0.59)	0.10 (0.02, 0.55)
Grading	G1 or G2	35	34	5.0 (3.1, 11.1)	0.46 (0.32, 0.65)	0.28 (0.17, 0.48)	0.09 (0.03, 0.27)
	G3	17	15	4.7 (2.0, NA)	0.38 (0.20, 0.71)	0.19 (0.07, 0.52)	0.19 (0.07, 0.52)
Line of Therapy	2	21	17	9.4 (5.8, 34.9)	0.65 (0.47, 0.89)	0.45 (0.27, 0.73)	0.23 (0.09, 0.58)
	3 or 4	20	20	4.6 (3.8, 12.3)	0.45 (0.28, 0.73)	0.30 (0.15, 0.59)	0.10 (0.03, 0.37)
	> 4	16	16	2.4 (1.7, 9.3)	0.25 (0.11, 0.58)	0.06 (0.01, 0.42)	0.06 (0.01, 0.42)
Metastasis timing	De novo metastasis	15	14	8.4 (4.6, 28.2)	0.57 (0.36, 0.90)	0.29 (0.12, 0.65)	0.21 (0.08, 0.58)
	Metastasis ≤ 60 months	12	10	4.0 (2.3, NA)	0.42 (0.21, 0.81)	0.33 (0.15, 0.74)	0.00
	Metastasis > 60 month	24	23	4.7 (2.8, 12.3)	0.45 (0.29, 0.70)	0.33 (0.19, 0.58)	0.14 (0.05, 0.40)
Concomitant diseases	0	10	9	11.7 (4.7, NA)	0.70 (0.47, 1.00)	0.50 (0.27, 0.93)	0.38 (0.16, 0.87)
	1—2	30	28	5.9 (2.8, 12.2)	0.48 (0.33, 0.70)	0.31 (0.18, 0.53)	0.12 (0.04, 0.33)
	≥ 3	17	16	3.8 (2.3, 9.3)	0.29 (0.14, 0.61)	0.12 (0.03, 0.43)	0.00
Diabetes	Yes	8	8	5.3 (4.6, NA)	0.38 (0.15, 0.92)	0.12 (0.02, 0.78)	0.12 (0.02, 0.78)
	No	45	42	4.6 (2.7, 9.5)	0.45 (0.33, 0.63)	0.27 (0.17, 0.44)	(0.05, 0.27)
Prior CDK4/6i therapy	Yes	55	52	5.0 (3.1, 9.4)	0.46 (0.35, 0.62)	0.28 (0.18, 0.43)	0.13 (0.06, 0.26)
PIK3CA mutation	p.E542K	9	9	2.1 (1.8, NA)	0.33 (0.13, 0.84)	0.22 (0.07, 0.75)	0.22 (0.07, 0.75)
	p.H1047R	16	16	6.0 (3.1, 24.1)	0.50 (0.31, 0.82)	0.44 (0.25, 0.76)	0.19 (0.07, 0.52)
	p.E545K	12	11	7.4 (2.3, NA)	0.57 (0.35, 0.94)	0.25 (0.09, 0.66)	0.00^2^
	Other	10	8	2.8 (1.7, NA)	0.33 (0.13, 0.84)	0.00^1^	0.00^2^
	p.E542K	9	9	2.1 (1.8, NA)	0.33 (0.13, 0.84)	0.22 (0.07, 0.75)	0.22 (0.07, 0.75)
Duration of first line CDK4/6 therapy^3^	< 24 months	12	10	9.0 (1.9, NA)	0.62 (0.39, 0.99)	0.27 (0.10, 0.71)	0.13 (0.02, 0.73)
	≥ 24 months	8	7	13.6 (5.8, NA)	0.62 (0.37, 1.00)	0.62 (0.37, 1.00)	0.31 (0.10, 0.96)

*CI* confidence interval, *BMI* body mass index ^1^No patient reached an observation time of 12 months ^2^No patient reached an observation time of 24 months ^3^Exploratory subgroup analysis for patients receiving first line CDK4/6i therapy directly followed by second line alpelisib plus fulvestrant (N = 20 patients)

Median PFS for patients pretreated with CDK4/6i therapy was 5.0 months (95% CI, 3.1–9.4). Subgroups were displayed exploratively, and, due to small subgroup size, no statistical testing was performed. Under these restrictions, it appeared that a higher number of previous therapies in the metastatic setting and a higher number of concomitant diseases had a negative impact on PFS; PFS was numerically highest in patients without concomitant diseases (concomitant diseases at the start of the alpelisib line are listed in the supplementary section, Table S6), at 11.7 months (95% CI, 4.7–NA), and lowest when alpelisib and fulvestrant were administered from the fourth line of therapy onwards, at 2.4 months (95% CI, 1.7–9.3) (Table 2). The number of patients at the start of the observation (N = 56) was lower than the actual patients included (N = 57) due to left truncation.

### Overall survival

**Fig. 2:**
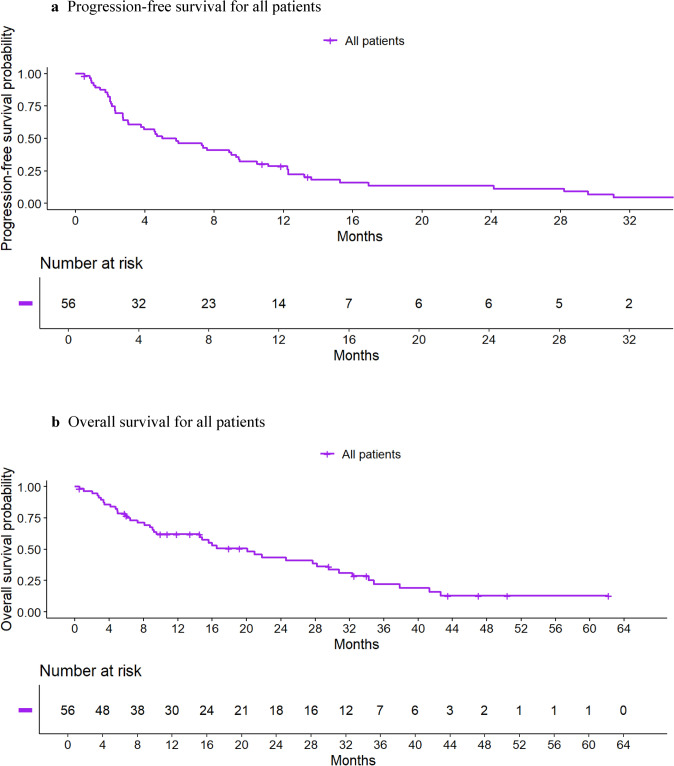
Progression-free (2a) and overall survival (2b) for all patients (N = 57). **a** Progression-free survival for all patients. **b** Overall survival for all patients

**Fig. 3:**
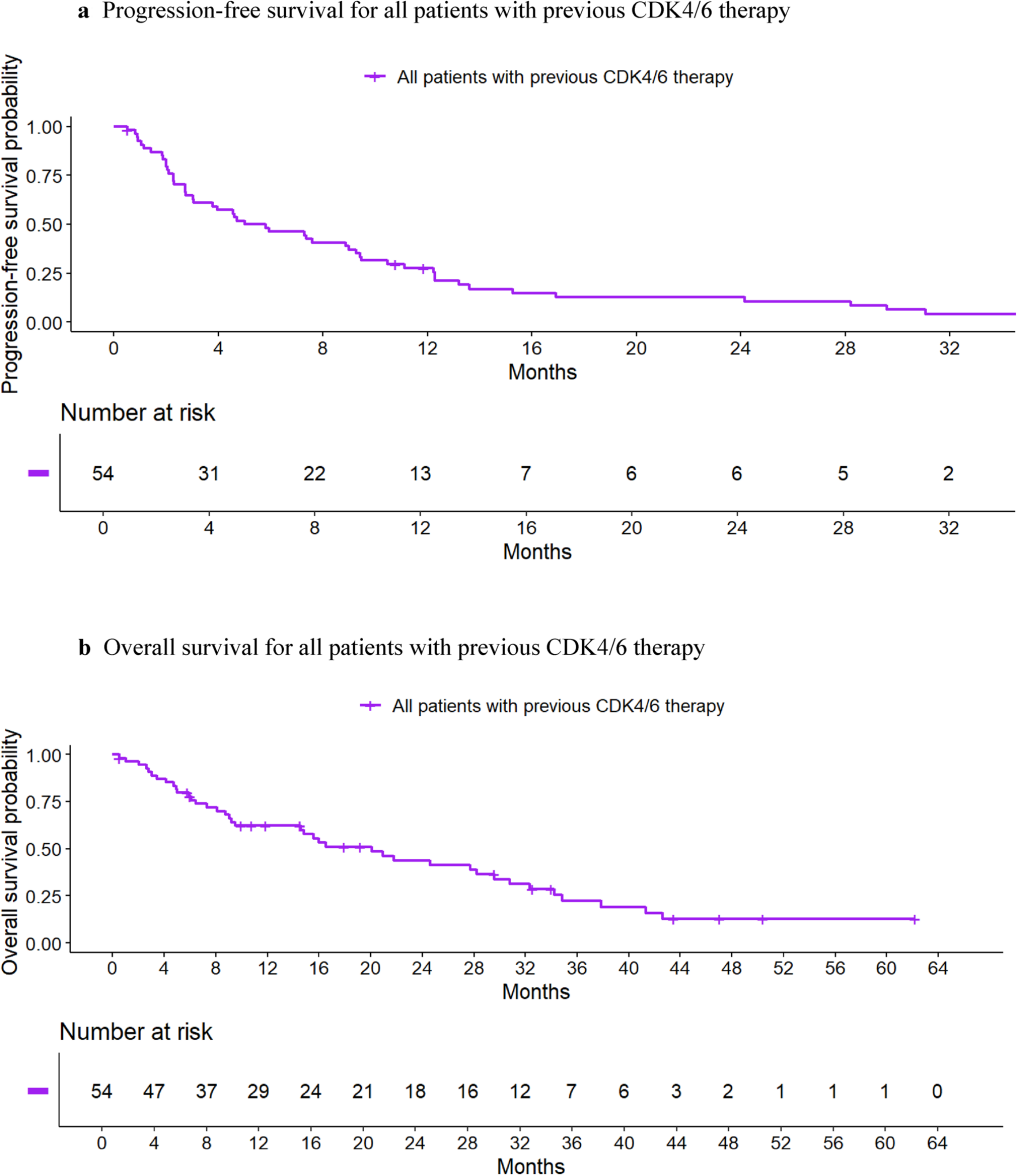
Progression-free survival (3a) and overall survival (3b) for all patients with previous CDK4/6 therapy (N = 55). **a** Progression-free survival for all patients with previous CDK4/6 therapy. **b** Overall survival for all patients with previous CDK4/6 therapy

The median OS time was 20.1 months (95% CI, 14.6–30.8) for all patients and 20.1 months (95% CI, 14.6–30.8) for those receiving prior CDK4/6i therapy. Figure 2 presents the Kaplan–Meier survival graphs for PFS and OS for all patients and Fig. 3 for those with prior CDK4/6i therapy.

**Fig. 4:**
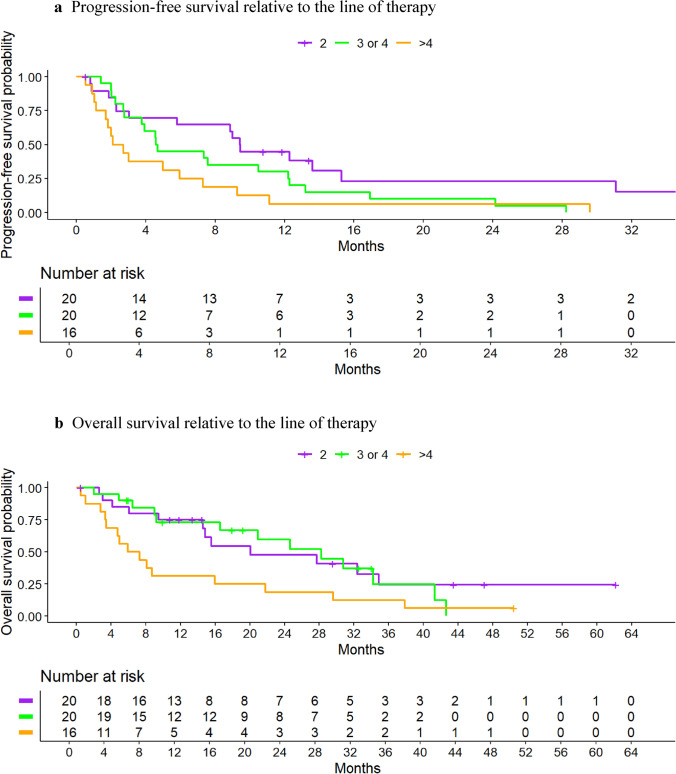
Progression-free (4a) and overall survival (4b) for all patients relative to the line of therapy. **a** Progression-free survival relative to the line of therapy. **b** Overall survival relative to the line of therapy

Subgroups were displayed exploratively, and, due to the small subgroups, no statistical testing was performed. Under these limitations, patients with de novo metastasis (median OS: 28.2 months [95% CI, 14.6–NA]) appeared to have longer median OS, while a higher therapy line (fourth line of therapy or later, 6.6 months [95% CI, 3.4–29.6]) was linked to shorter OS (Table 3). Figure 4 presents the Kaplan–Meier curves for PFS and OS regarding line of therapy.

**Table 3:** Median overall survival times and survival rates

Outcome		N	Events	Median survival time^1^ (95% CI) in months	6-month survival rate (95% CI)	12-month survival rate (95% CI)	24-month survival rate (95% CI)
All patients		57	40	20.1 (14.6, 30.8)	0.77 (0.66, 0.89)	0.62 (0.50, 0.76)	0.43 (0.31, 0.60)
Age	Up to 49	3	2	16.0 (5.0, NA)	0.67 (0.30, 1.00)	0.67 (0.30, 1.00)	0.00
	50—74	45	32	15.6 (9.0, 32.4)	0.75 (0.63, 0.89)	0.57 (0.44, 0.73)	0.43 (0.30, 0.61)
	75 +	9	6	24.6 (16.6, NA)	0.89 (0.71, 1.00)	0.89 (0.71, 1.00)	0.59 (0.32, 1.00)
BMI	Normal	32	23	20.1 (9.5, 34.2)	0.78 (0.64, 0.94)	0.64 (0.49, 0.84)	0.49 (0.33, 0.71)
	Overweight/Obese	21	14	20.9 (8.1, NA)	0.81 (0.66, 1.00)	0.61 (0.43, 0.86)	0.39 (0.21, 0.72)
ECOG	0	37	27	16.0 (9.2, 34.2)	0.78 (0.66, 0.93)	0.59 (0.45, 0.77)	0.45 (0.31, 0.66)
	1 or 2	19	12	20.1 (15.6, NA)	0.78 (0.61, 1.00)	0.72 (0.54, 0.96)	0.41 (0.22, 0.77)
Grading	G1 or G2	35	27	20.1 (9.0, 30.8)	0.71 (0.58, 0.88)	0.57 (0.43, 0.76)	0.40 (0.26, 0.61)
	G3	17	10	14.9 (6.5, NA)	0.82 (0.65, 1.00)	0.59 (0.39, 0.91)	0.40 (0.19, 0.80)
Line of Therapy	2	21	12	20.1 (14.6, NA)	0.85 (0.71, 1.00)	0.75 (0.58, 0.97)	0.48 (0.29, 0.80)
	3 or 4	20	13	28.2 (16.6, NA)	0.90 (0.78, 1.00)	0.73 (0.55, 0.96)	0.60 (0.40, 0.89)
	> 4	16	15	6.6 (3.4, 29.6)	0.50 (0.31, 0.82)	0.31 (0.15, 0.65)	0.19 (0.07, 0.52)
Metastasis timing	De novo metastasis	15	11	28.2 (14.6, NA)	0.86 (0.69, 1.00)	0.78 (0.59, 1.00)	0.61 (0.39, 0.95)
	Metastasis ≤ 60 months	12	7	12.7 (5.0, NA)	0.67 (0.45, 0.99)	0.50 (0.28, 0.88)	0.33 (0.13, 0.89)
	Metastasis > 60 month	24	16	21.8 (9.2, NA)	0.75 (0.59, 0.94)	0.66 (0.49, 0.88)	0.46 (0.29, 0.73)
Concomitant diseases	0	10	8	16.0 (14.6, NA)	0.90 (0.73, 1.00)	0.80 (0.59, 1.00)	0.46 (0.22, 0.93)
	1—2	30	19	15.6 (7.3, NA)	0.69 (0.54, 0.88)	0.54 (0.39, 0.76)	0.50 (0.34, 0.73)
	≥ 3	17	13	20.1 (9.2, NA)	0.82 (0.66, 1.00)	0.63 (0.44, 0.92)	0.32 (0.15, 0.70)
Diabetes	Yes	8	7	9.0 (6.5, NA)	0.88 (0.67, 1.00)	0.44 (0.19, 1.00)	0.15 (0.02, 0.89)
	No	45	30	21.8 (15.6, 34.2)	0.75 (0.63, 0.89)	0.63 (0.50, 0.79)	0.49 (0.35, 0.67)
Prior CDK4/6i therapy	Yes	55	39	20.1 (14.6, 30.8)	0.78 (0.67, 0.90)	0.62 (0.50, 0.77)	0.44 (0.32, 0.61)
PIK3CA mutation	p.E542K	9	8	16.0 (8.1, NA)	0.78 (0.55, 1.00)	0.52 (0.27, 1.00)	0.39 (0.16, 0.93)
	p.H1047R	16	12	20.1 (6.5, NA)	0.75 (0.57, 1.00)	0.62 (0.43, 0.91)	0.40 (0.21, 0.76)
	p.E545K	12	7	27.7 (2.8, NA)	0.66 (0.44, 0.99)	0.58 (0.36, 0.94)	0.58 (0.36, 0.94)
	Other	10	7	21.8 (9.0, NA)	0.89 (0.71, 1.00)	0.67 (0.42, 1.00)	0.40 (0.17, 0.94)
	p.E542K	9	8	16.0 (8.1, NA)	0.78 (0.55, 1.00)	0.52 (0.27, 1.00)	0.39 (0.16, 0.93)
Duration of first line CDK4/6 therapy^2^	< 24 months	12	9	15.6 (14.6, NA)	0.82 (0.62, 1.00)	0.73 (0.51, 1.00)	0.31 (0.12, 0.79)
	≥ 24 months	8	3	NA (32.4, NA)	0.88 (0.67, 1.00)	0.75 (0.50, 1.00)	0.75 (0.50, 1.00)

*CI* confidence interval, *BMI* body mass index^1^No patient reached an observation time of 24 month^2^Exploratory subgroup analysis for patients receiving first line CDK4/6i therapy directly followed by second line alpelisib plus fulvestrant (N = 20 patients)

Further PFS and OS Kaplan–Meier graphs based on different cofactors (age, metastasis timing, concomitant diseases, BMI, ECOG, tumor grading, metastasis sites, preexisting diabetes, PIK3CA mutation and duration of first-line CDK4/6 therapy in patients receiving second line alpelisib) are presented in the supplementary section (Fig. S5a–j for PFS and Fig. S6a–j for OS).

### Serious adverse and adverse events

In the summary of all AEs by the Medical Dictionary for Regulatory Activities Preferred Terms (MedDRA PT), hyperglycemia was the most frequently reported AE, occurring in 22.8% (N = 13) of patients, followed by skin toxicity in 14% (N = 8), and diarrhea in 10.5% (N = 6) of patients (Table S7, supplementary section). Hyperglycemia was the most frequently mentioned specific Grade 3 or 4 AE occurring in 16.7% (N = 2) of patients (Table S8 supplementary section). Dyspnea (N = 3; 12%), lung infection (N = 2; 8%) and upper respiratory infection (N = 2; 8%) were the most frequently mentioned specific serious AEs, leading to hospitalization (Table S9supplementary section).

## Discussion

In this real-world population with patients having advanced HRpos, HER2neg, *PIK3CA-*mutated breast cancer, median PFS (5.0 months) and OS (20.1 months) for patients treated with alpelisib were slightly shorter than those reported in pivotal trials. Compared with these trials, patients were more heavily pretreated (with 63.1% receiving alpelisib in the third line or beyond). Almost all patients (55 of 57) received prior CDK4/6i therapy.

Alpelisib was regularly available in Germany between July 27, 2020 and April 30, 2021. Thereafter, the drug was only useable after an approved cost coverage application and individual import from another EU country. The reason was a lack of compromise on pricing and reimbursement between the manufacturer and health insurance representatives following the benefit assessment of the drug (significant improvement in PFS, but no significant improvement in OS in the pivotal SOLAR-1 study). Assuming a *PIK3CA* mutation frequency of 40% in the HRpos HER2neg cohort and subtracting the patients, who died before approval, approximately 850 patients from the PRAEGNANT cohort would have had a treatment option with alpelisib irrespective of clinical studies before alpelisib was approved. Beside limited access, challenging management of adverse events, most notably hyperglycemia, preference for non-endocrine-based therapy and death in the first line of therapy for ABC likely reduced actual use. Between 2020 and 2022 13.6 to 16.7% of patients received chemotherapy as the first line treatment [27], furthermore, around 15% of HRpos HER2neg patients died in the first line of therapy [28]. Adjusting for these factors, 600 patients remained in our dataset potentially eligible to receive alpelisib. Approximately 10% of patients during the study period received alpelisib therapy, despite clear therapeutic indications, regulatory approval, and guideline recommendations. These guideline recommendations also apply in particular to second-line treatment with prior treatment with a CDK4/6i [29, 30]. Notably, the phase III EPIK-B5 trial specifically evaluated alpelisib plus fulvestrant in patients with PIK3CA-mutated HR + HER2 − ABC after progression on an aromatase inhibitor and a CDK4/6i, providing randomized evidence for this clinically common sequence; primary analyses reported a significant PFS benefit [31]. The observed shortfall may in part reflect reimbursement denials by health insurers, despite treatment recommendations made during tumor board discussions. This indicates the substantial potential for treating HRpos, HER2neg, *PIK3CA*-mutated breast cancer patients with a targeted therapy.

Patients in the SOLAR-1 study received alpelisib at an earlier line of treatment and PFS as well as OS were longer compared to our study cohort [8, 12]. The mutations described were comparable to our cohort. The same was true for AEs, which were described more frequently in the SOLAR-1 study.

Compared with our cohort, patients in the BYLieve study received alpelisib earlier as well [13]. However, all patients also received CDK4/6i as prior therapy. Median PFS and OS were slightly longer than in our cohort, but shorter than in the SOLAR-1 study. While PIK3CA mutations were not reported, adverse events were similar. However, they were documented considerably more frequently compared to our analysis.

Patients in the French EAP were even more heavily pre-treated than our cohort and had a shorter median OS, despite a similar PFS [15]. The side effects described were comparable and slightly more frequent than in our cohort.

It appears that the differences in PFS and OS might be explained by the timing of alpelisib treatment. Administration of alpelisib in an earlier line of therapy, such as lines 1 and 2, as in the SOLAR-1 trial, leads to longer PFS and OS times. In contrast, the heavily pre-treated patients in the French EAP had the shortest PFS and OS, leaving BYLieve between the other two studies in terms of both prior therapies and PFS and OS times.

The present findings suggest a markedly longer PFS in patients receiving alpelisib and fulvestrant in the absence of additional comorbidities at the initiation of therapy, but sample size is a limitation. In contrast, PFS seemed to be shortest in the subgroup of patients with three or more comorbidities, diabetes, arterial hypertension and pain due to several causes were the most commonly reported conditions. Interestingly, the number of concomitant diseases did not appear to affect OS. We performed a more detailed analysis of patients with diabetes (N = 8). In this subgroup, in contrast to the overall burden of comorbidities, the main impact was observed on overall survival (OS), whereas progression-free survival (PFS) was formally similar between the two groups (Figs. 5h and 6 h).

Neither SOLAR-1, BYLIEVE, nor the French EAP addressed the total number of comorbidities, so comparisons with the studies are not useful in this regard. Our evaluation therefore adds new insights here.

A growing body of evidence shows that comorbidity burden indices such as the Charlson Comorbidity Index (CCI) are a powerful and independent determinant of OS in women with ABC [32]. Importantly, the adverse impact of a high CCI persists after accounting for aggressive tumor biology, such as triple-negative disease, suggesting that optimal management of concomitant conditions is a modifiable lever to improve equity as well as longevity [33]. However, based on the data presented here, the number of comorbidities at the start of therapy only affected PFS, not OS. The lack of impact on overall survival is not entirely clear to us, but it likely reflects the heterogeneity of the study population and suggests that comorbidities account for only part of the overall prognosis. In contrast, patients with diabetes exhibited a worse overall survival.

This highlights the central importance of hyperglycemia as a potential side effect of the PI3CA-inhibitor alpelisib. Hyperglycemia is an inherent adverse effect of PI3CA pathway inhibition and becomes increasingly problematic as the extent of PI3CA inhibition broadens. Target-oriented side effect management is therefore a key component of successful alpelisib therapy from the very beginning. This includes, for example, prophylactic treatment with metformin to prevent hyperglycemia, as well as alpelisib dose reductions in the presence of elevated blood glucose levels [34].

Particularly noteworthy prognostic factors besides pre-existing diabetes is obesity and age above 75 years, which are explicitly mentioned in the drug’s approval. Interestingly, in our cohort, age above 75 appeared to be associated with a lower PFS but a higher OS. Overweight or obesity did not appear to affect PFS or OS in our small study cohort.

Patients who developed metastasis in the first 60 months after primary diagnosis exhibited the shortest PFS and OS, whereas those with de novo disease showed the longest survival times. The same was observed in all evaluable HRpos, HER2neg patients with follow-up data from the PRAEGNANT registry, regardless of the therapy received [35].

A comparison of the median PFS of our cohort with capivasertib and fulvestrant (CAPItello-291 study, [6]), a recently approved treatment option for patients in a slightly broader therapeutic indication (HRpos HER2neg ABC with mutations in the *AKT1* pathway, *PIK3CA, AKT1, PTEN*), shows similar median PFS (7.3 months in the *AKT1* pathway-altered group). While highly exploratory, such comparisons help to assess therapy effects.

The recently approved combination of the *PIK3CA* inhibitor inavolisib with palbociclib, and fulvestrant demonstrated an impressive median PFS with 17.2 months (95% CI, 11.6–22.2) in 161 patients who received the triplet in the pivotal INNAVO 120 trial [7]. ABC patients that progressed during or within 12 months after completion of adjuvant endocrine therapy were enrolled. These patients had no previous therapy in the metastatic setting, making them, on the one hand, susceptible to first-line metastatic treatment. On the other hand, these endocrine-resistant patients belong to a group with a very poor prognosis.

The main limitation of our study analysis is the small sample size, both in the overall population and even more so concerning subgroup analysis. Therefore, all subgroup comparisons should be considered as purely hypothesis generating. In this study we generated RWD for the use of alpelisib, which, alongside the EAP in France, represents the most extensive global real-world analysis to date and broadly confirms previously published PFS and OS data, as well as the frequency of *PIK3CA* mutations and reported AEs. In addition, our evaluation provides accurate subgroup analyses, which, however, can only be considered exploratory due to the small group sizes.

## Conclusion

This prospective real-world analysis from the PRAEGNANT registry shows slightly shorter PFS and OS as comparative trials with exploratory subgroups analysis. Receiving alpelisib in earlier therapy lines, de novo metastatic disease, and fewer concomitant diseases were associated with numerically better PFS although subgroups were too small to draw statistically significant conclusions. Receiving alpelisib in later therapy lines and pre-treatment diabetes seems to be linked to shorter OS. In this RWD analysis, AEs were less often documented than in the pivotal studies, possibly related to underreporting. Discontinuation of therapy due to toxicity was still relatively common, but less frequent than in the approval studies, possibly due to improved side effect management, patient selection, and clinical experiences gained from both SOLAR-1 and BYLieve trials. The most often reported AEs in this trial were hyperglycemia, diarrhea and rash. The evaluation offers additional evidence supporting the benefit of alpelisib and fulvestrant after CDK4/6i in patients with HRpos HER2neg ABC. It also provides a RWD analysis as a comparator for new *PIK3CA/AKT1/PTEN* pathway therapies such as capivasertib or inavolisib.

## Supplementary Information

Below is the link to the electronic supplementary material.

Supplementary file1 (DOCX 472 KB)

## Data Availability

The datasets used and analyzed during the current study are available upon reasonable request in the context of a research project. Proposals are evaluated by the PRAEGNANT scientific board. In case of approval, data is available from the corresponding author.

## Abbreviations

ABC: Advanced breast cancer
CDK4/6i: Cyclin-dependent kinases 4 and 6 inhibitor
EAP: Early Access Program
ECOG: Eastern Cooperative Oncology Group performance status
EMA: European Medicines Agency
ESR1: Estrogen receptor 1
FDA: Food and Drug Administration
HER2: Human epidermal growth factor receptor 2
HER2neg: Human epidermal growth factor receptor 2-negative
HR: Hormone receptor
HRpos: Hormone receptor positive
OS: Overall survival
PI3K: Phosphatidylinositol 3-kinase
PIK3CA: Phosphatidylinositol-4,5-bisphosphate 3-kinase Catalytic Subunit Alpha
PFS: Progression-free survival
RWD: Real-world data
(S)AE: (Serious) Adverse event
