# Supplementary material for: Alpelisib and Fulvestrant in PIK3CA-mutated hormone receptor-positive HER2-negative advanced breast cancer included in the German PRAEGNANT trial
Source: Breast Cancer Res Treat. 2026 Apr 2;217(1):9. doi: 10.1007/s10549-026-07939-z (PMC13046609; doi:10.1007/s10549-026-07939-z)

Supplementary Material

**Table 4:** Frequency table with the specific *PIK3CA* mutations, showing frequency and percentage (N = 57 patients)

| **PIK3CA mutations** | **PIK3CA mutation classification for PFS and OS analyses** | **N (%)** |
| --- | --- | --- |
| p.C420R | other | 1 (1.8) |
| p.C420R;p.H1047R | p.H1047R | 1 (1.8) |
| p.E542K | p.E542K | 9 (15.8) |
| p.E542K;p.E545K | p.E545K | 1 (1.8) |
| p.E545K | p.E545K | 10 (17.5) |
| p.E545K;p.A1066V | p.E545K | 1 (1.8) |
| p.G451V | other | 2 (3.5) |
| p.H1047L | other | 3 (5.3) |
| p.H1047R | p.H1047R | 13 (22.8) |
| p.H1047R;p.E726K | p.H1047R | 1 (1.8) |
| p.H1047R;R115L | p.H1047R | 1 (1.8) |
| p.N345K | other | 1 (1.8) |
| p.R524K | other | 2 (3.5) |
| p.V344G;p.H1047L | other | 1 (1.8) |
| Unknown | Unknown | 10 (17.5) |

**Table 5:** Type of therapy administered after Alpelisib (N = 36)

|  | N (%) |
| --- | --- |
| Antibody-drug conjugate | 3 (8.3) |
| Chemo | 19 (52.8) |
| Endocrine therapy + Everolimus | 8 (22.2) |
| Endocrine therapy other | 2 (5.6) |
| Other | 4 (11.1) |

**Table 6:** Concomittant diseases at start of alpelisib line

| **Concommittant disease** | **N (%)** |
| --- | --- |
| other | 19 (33.3) |
| diabetes mellitus | 8 (14.0) |
| arterial hypertension | 7 (12.3) |
| pain | 4 (7.0) |
| hypothyroidism | 3 (5.3) |
| lymphedema | 3 (5.3) |
| depression | 3 (5.3) |
| concordant cancer | 2 (3.5) |
| coronary heart disease (CHD) | 2 (3.5) |
| paroxysmal atrial fibrillation | 2 (3.5) |
| pulmonary embolism | 1 (1.8) |
| pulmonary fibrosis | 1 (1.8) |
| thromboses | 1 (1.8) |
| nausea | 1 (1.8) |
| osteoporosis | 1 (1.8) |
| cataract | 1 (1.8) |
| anxiety disorder | 1 (1.8) |
| diarrhea | 1 (1.8) |
| glaucoma | 1 (1.8) |

**Table 7** Summary of all adverse events (AEs) by Medical Dictionary for Regulatory Activities (MedDRA) system organ class (SOC) and preferred term (PT). Percentages refer to the total numbers of events (N = 79 events) and patients (N = 57 patients)^1^

| **MedDRA SOC** | **MedDRA PT** | **Events N (%)** | **Patients N (%)** |
| --- | --- | --- | --- |
| Blood and lymphatic system disorders | Anemia | 1 (1.3) | 1 (1.8) |
| Gastrointestinal disorders | Abdominal pain | 2 (2.5) | 2 (3.5) |
| Gastrointestinal disorders | Diarrhea | 6 (7.6) | 6 (10.5) |
| Gastrointestinal disorders | Gastritis | 1 (1.3) | 1 (1.8) |
| Gastrointestinal disorders | Mucositis oral | 4 (5.1) | 4 (7.0) |
| Gastrointestinal disorders | Nausea | 3 (3.8) | 3 (5.3) |
| Gastrointestinal disorders | Vomiting | 1 (1.3) | 1 (1.8) |
| General disorders and administration site conditions | Disease progression | 1 (1.3) | 1 (1.8) |
| General disorders and administration site conditions | Fatigue | 1 (1.3) | 1 (1.8) |
| General disorders and administration site conditions | General disorders and administration site conditions - Other, specify | 6 (7.6) | 4 (7.0) |
| Infections and infestations | Bronchial infection | 1 (1.3) | 1 (1.8) |
| Infections and infestations | Lung infection | 2 (2.5) | 1 (1.8) |
| Infections and infestations | Pharyngitis | 1 (1.3) | 1 (1.8) |
| Infections and infestations | Shingles | 1 (1.3) | 1 (1.8) |
| Infections and infestations | Upper respiratory infection | 3 (3.8) | 2 (3.5) |
| Infections and infestations | Urinary tract infection | 2 (2.5) | 2 (3.5) |
| Infections and infestations | Vaginal infection | 1 (1.3) | 1 (1.8) |
| Injury, poisoning and procedural complications | Fracture | 1 (1.3) | 1 (1.8) |
| Injury, poisoning and procedural complications | Injury, poisoning and procedural complications - Other, specify | 1 (1.3) | 1 (1.8) |
| Investigations | Creatinine increased | 1 (1.3) | 1 (1.8) |
| Investigations | Neutrophil count decreased | 1 (1.3) | 1 (1.8) |
| Investigations | Platelet count decreased | 1 (1.3) | 1 (1.8) |
| Investigations | White blood cell decreased | 1 (1.3) | 1 (1.8) |
| Metabolism and nutrition disorders | Anorexia | 1 (1.3) | 1 (1.8) |
| Metabolism and nutrition disorders | Hyperglycemia | 14 (17.7) | 13 (22.8) |
| Nervous system disorders | Headache | 1 (1.3) | 1 (1.8) |
| Psychiatric disorders | Agitation | 1 (1.3) | 1 (1.8) |
| Reproductive system and breast disorders | Vaginal dryness | 1 (1.3) | 1 (1.8) |
| Respiratory, thoracic and mediastinal disorders | Dyspnea | 3 (3.8) | 3 (5.3) |
| Respiratory, thoracic and mediastinal disorders | Epistaxis | 1 (1.3) | 1 (1.8) |
| Respiratory, thoracic and mediastinal disorders | Respiratory failure | 1 (1.3) | 1 (1.8) |
| Skin and subcutaneous tissue disorders | Nail changes | 1 (1.3) | 1 (1.8) |
| Skin and subcutaneous tissue disorders | Palmar-plantar erythrodysesthesia syndrome | 1 (1.3) | 1 (1.8) |
| Skin and subcutaneous tissue disorders | Pruritus | 1 (1.3) | 1 (1.8) |
| Skin and subcutaneous tissue disorders | Rash maculo-papular | 9 (11.4) | 8 (14.0) |
| Skin and subcutaneous tissue disorders | Skin and subcutaneous tissue disorders - Other, specify | 1 (1.3) | 1 (1.8) |

^1^ The table can be read as follows: For example, 14 hyperglycemia events were observed and these events occurred in 13 patients. In other words, there were 13 patients with one or more hyperglycemia events.

**Table 8** Summary of adverse events (AEs) of grade 3 or 4 by Medical Dictionary for Regulatory Activities (MedDRA) system organ class (SOC) and preferred term (PT). Percentages refer to the total numbers of events (N = 12 events) and patients (N = 57 patients)

| **MedDRA SOC** | **MedDRA PT** | **Events N (%)** | **Patients N (%)** |
| --- | --- | --- | --- |
| Gastrointestinal disorders | Abdominal pain | 1 (8.3) | 1 (1.8) |
| Gastrointestinal disorders | Diarrhea | 1 (8.3) | 1 (1.8) |
| Gastrointestinal disorders | Nausea | 1 (8.3) | 1 (1.8) |
| General disorders and administration site conditions | Disease progression | 1 (8.3) | 1 (1.8) |
| General disorders and administration site conditions | General disorders and administration site conditions - Other, specify | 2 (16.7) | 2 (3.5) |
| Metabolism and nutrition disorders | Hyperglycemia | 2 (16.7) | 2 (3.5) |
| Psychiatric disorders | Agitation | 1 (8.3) | 1 (1.8) |
| Respiratory, thoracic and mediastinal disorders | Dyspnea | 1 (8.3) | 1 (1.8) |
| Skin and subcutaneous tissue disorders | Pruritus | 1 (8.3) | 1 (1.8) |
| Skin and subcutaneous tissue disorders | Skin and subcutaneous tissue disorders - Other, specify | 1 (8.3) | 1 (1.8) |

**Table 9** Summary of all serious adverse events (SAEs) by Medical Dictionary for Regulatory Activities (MedDRA) system organ class (SOC) and preferred term (PT). Percentages refer to the total numbers of events (N = 25 events) and patients (N = 57 patients)

| **MedDRA SOC** | **MedDRA PT** | **Events N (%)** | **Patients N (%)** |
| --- | --- | --- | --- |
| Gastrointestinal disorders | Abdominal pain | 1 (4.0) | 1 (1.8) |
| Gastrointestinal disorders | Nausea | 1 (4.0) | 1 (1.8) |
| General disorders and administration site conditions | Disease progression | 1 (4.0) | 1 (1.8) |
| General disorders and administration site conditions | Fatigue | 1 (4.0) | 1 (1.8) |
| General disorders and administration site conditions | General disorders and administration site conditions - Other, specify | 6 (24.0) | 4 (7.0) |
| Infections and infestations | Lung infection | 2 (8.0) | 1 (1.8) |
| Infections and infestations | Pharyngitis | 1 (4.0) | 1 (1.8) |
| Infections and infestations | Upper respiratory infection | 2 (8.0) | 1 (1.8) |
| Infections and infestations | Urinary tract infection | 1 (4.0) | 1 (1.8) |
| Injury, poisoning and procedural complications | Injury, poisoning and procedural complications - Other, specify | 1 (4.0) | 1 (1.8) |
| Investigations | Creatinine increased | 1 (4.0) | 1 (1.8) |
| Investigations | Neutrophil count decreased | 1 (4.0) | 1 (1.8) |
| Metabolism and nutrition disorders | Anorexia | 1 (4.0) | 1 (1.8) |
| Psychiatric disorders | Agitation | 1 (4.0) | 1 (1.8) |
| Respiratory, thoracic and mediastinal disorders | Dyspnea | 3 (12.0) | 3 (5.3) |
| Respiratory, thoracic and mediastinal disorders | Respiratory failure | 1 (4.0) | 1 (1.8) |

**Figure 5** Progression-free survival relative to different parameters (age, body mass index, ECOG, tumor grading, metastasis pattern, metastasis timing, number of concomitant diseases, preexisting diabetes, PIK3CA mutation and duration of first-line CDK4/6 therapy in patients receiving second line Alpelisib)

**Figure 5a**: Progression-free survival relative to the age (years)


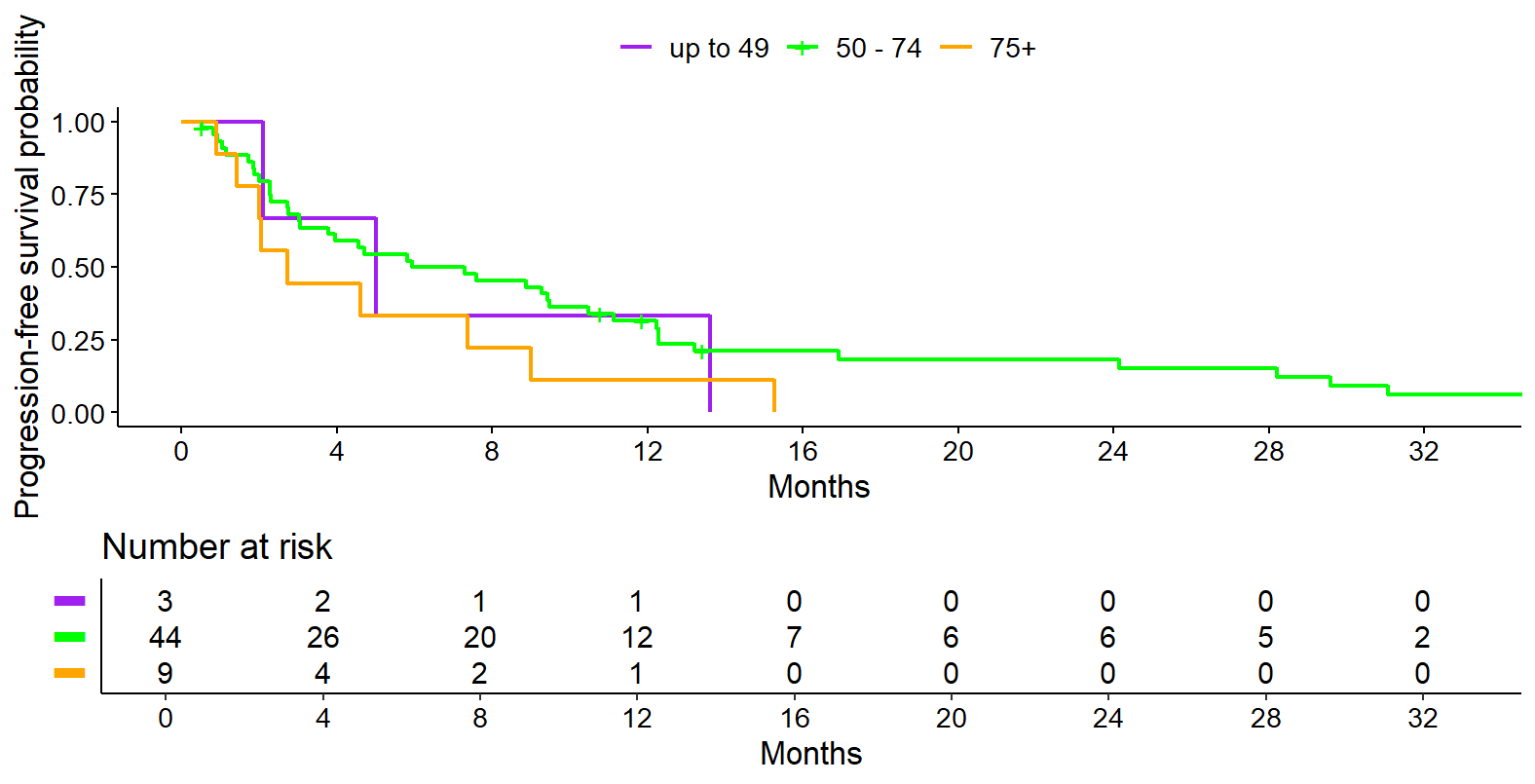


**Figure 5b**: Progression-free survival relative to the body mass index


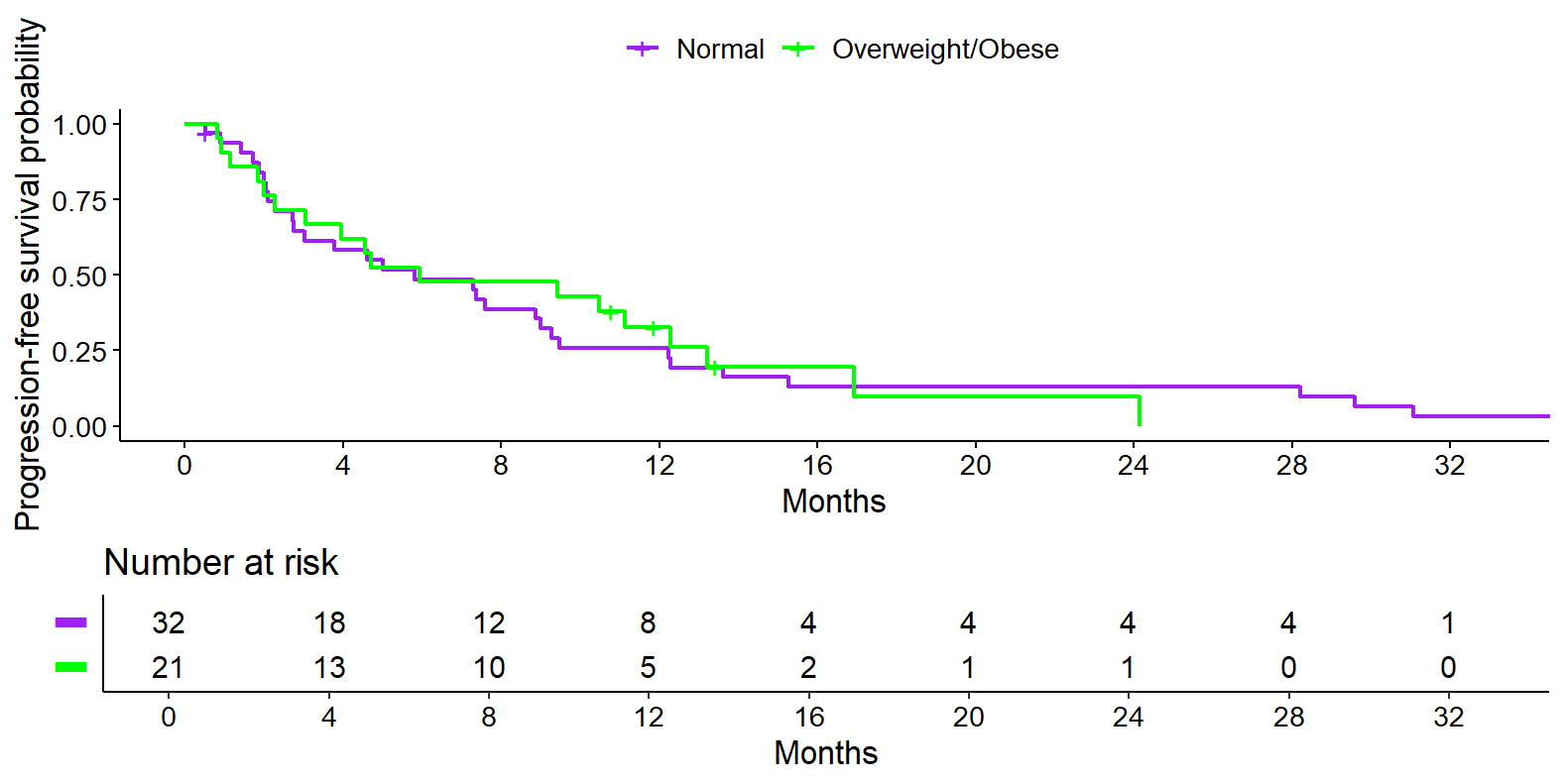


**Figure 5c**: Progression-free survival relative to the ECOG


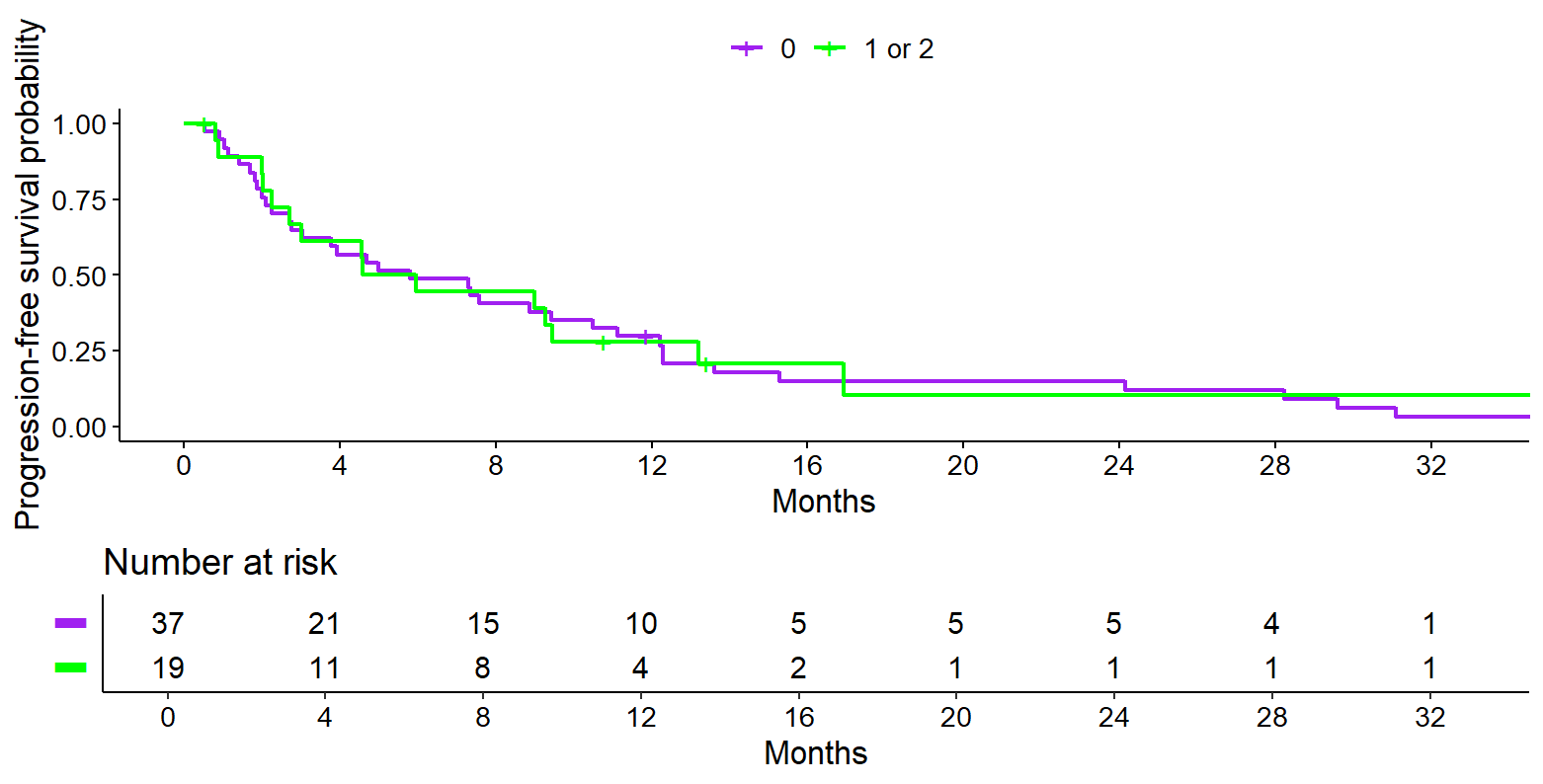


**Figure 5d**: Progression-free survival relative to the tumor grading


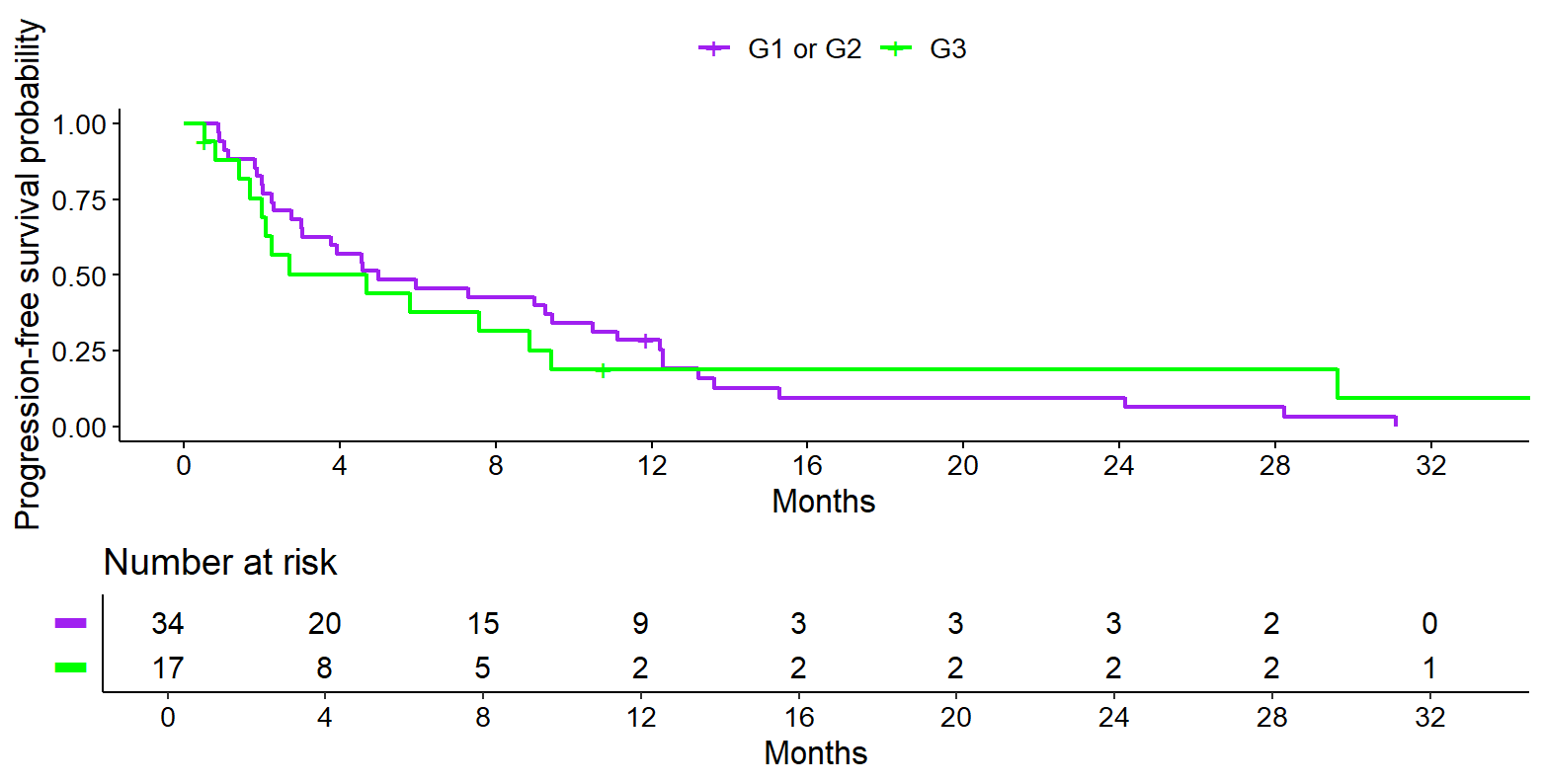


**Figure 5e**: Progression-free survival relative to metastasis pattern


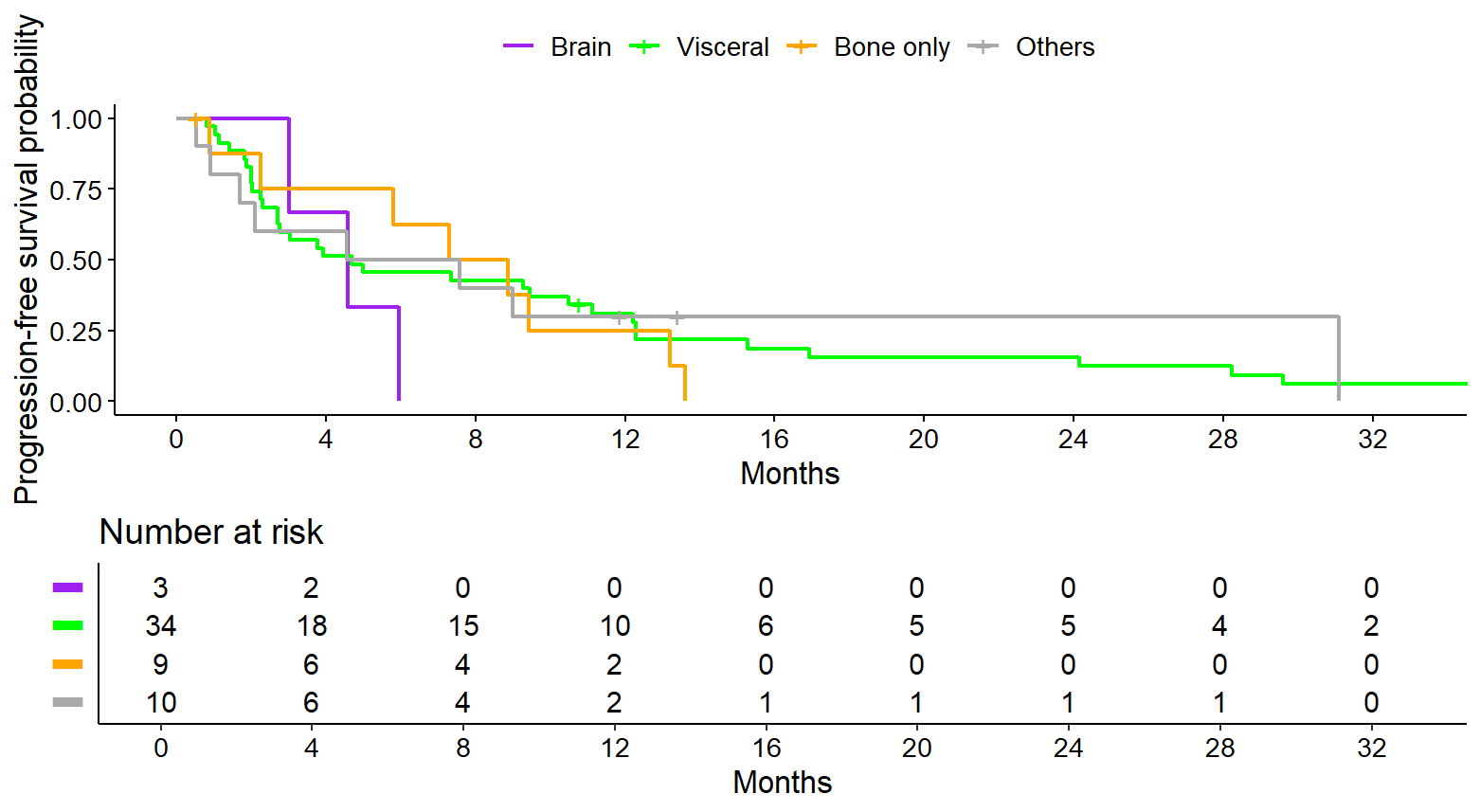


**Figure 5f**: Progression-free survival relative to metastasis timing (de novo, ≤ 60 months after primary diagnosis, > 60 months after primary diagnosis)


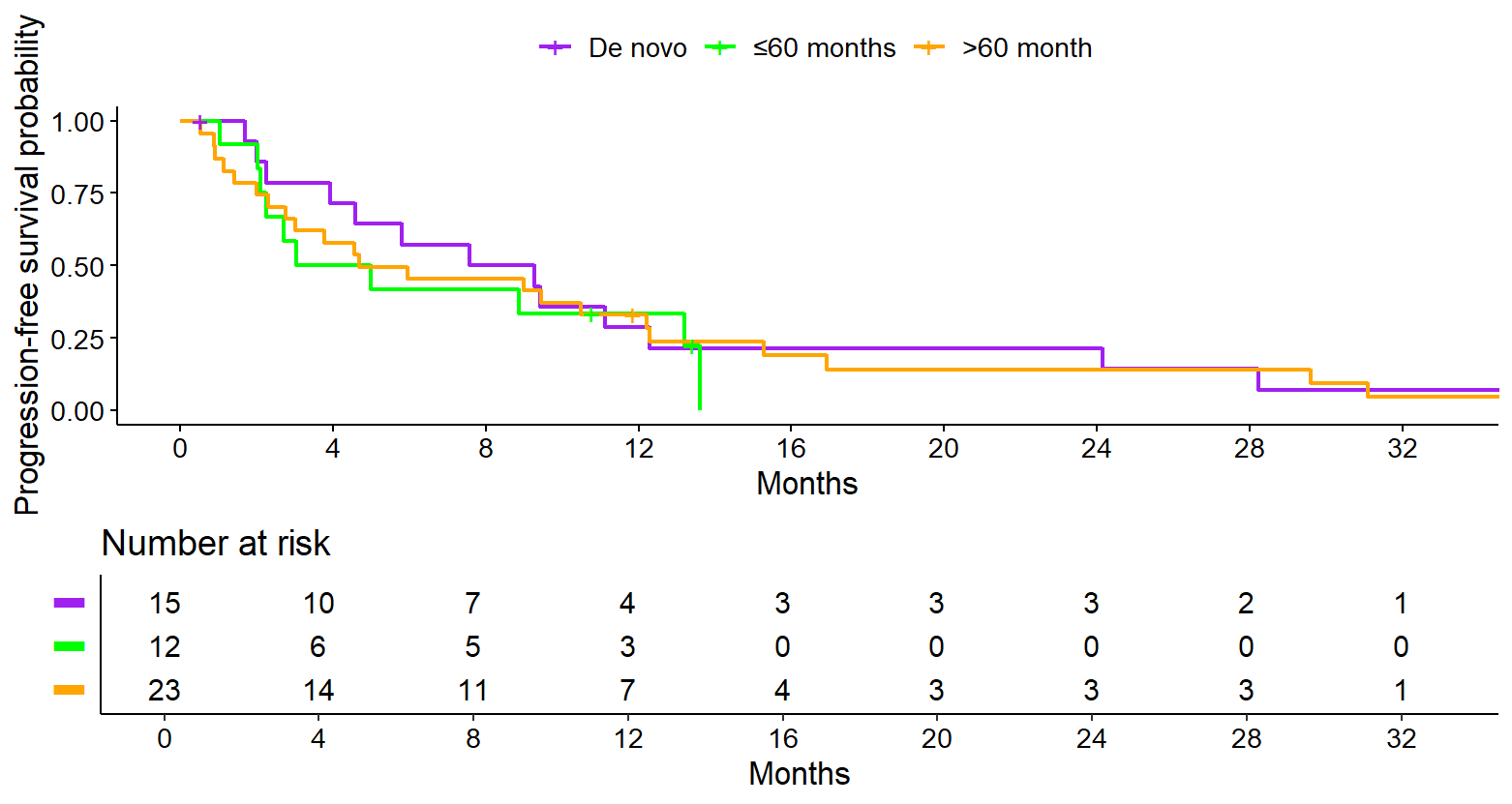


**Figure 5g**: Progression-free survival relative to the number of concomitant diseases


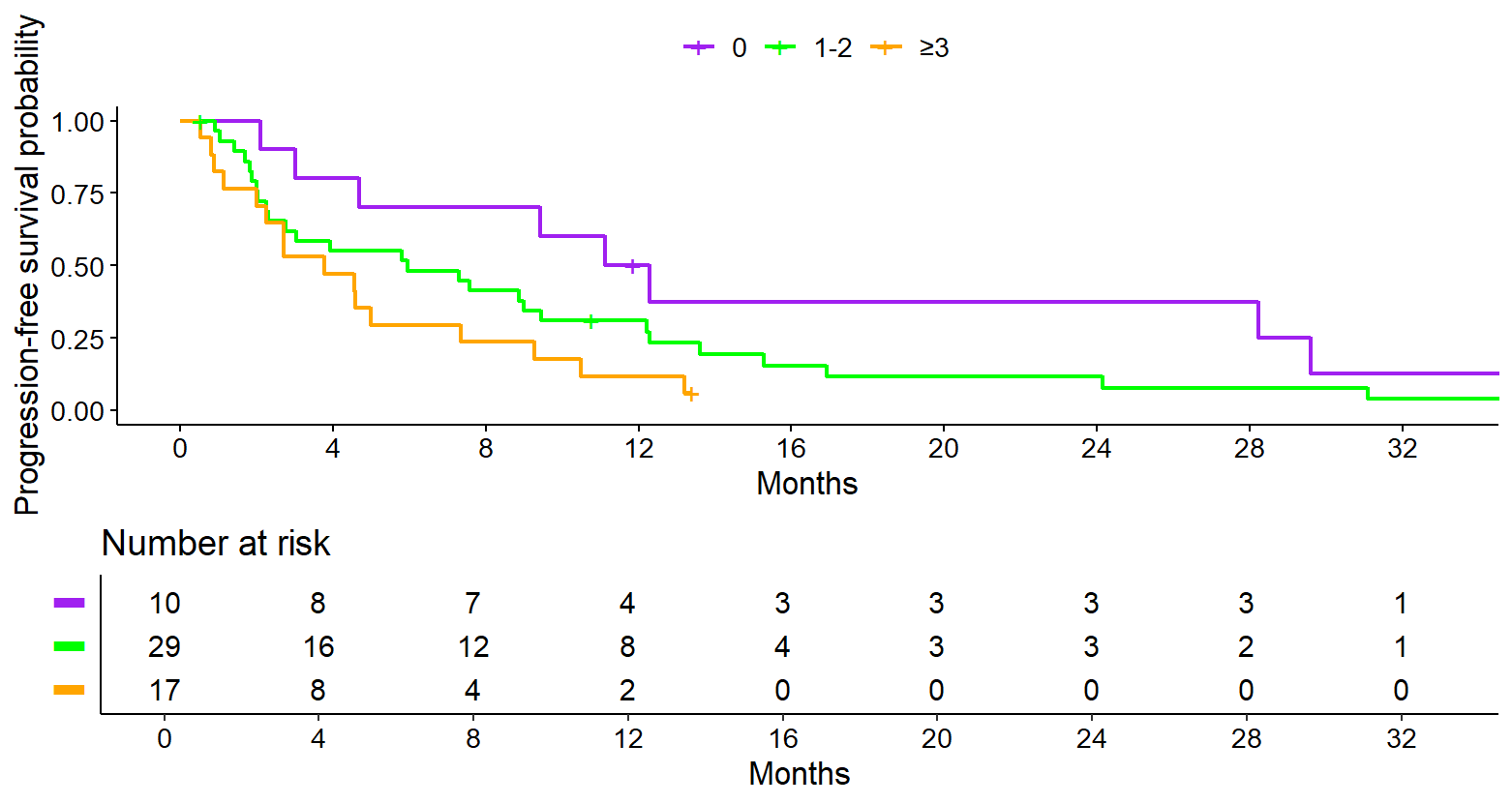


**Figure 5h**: Progression-free survival relative to diabetes (yes/no)


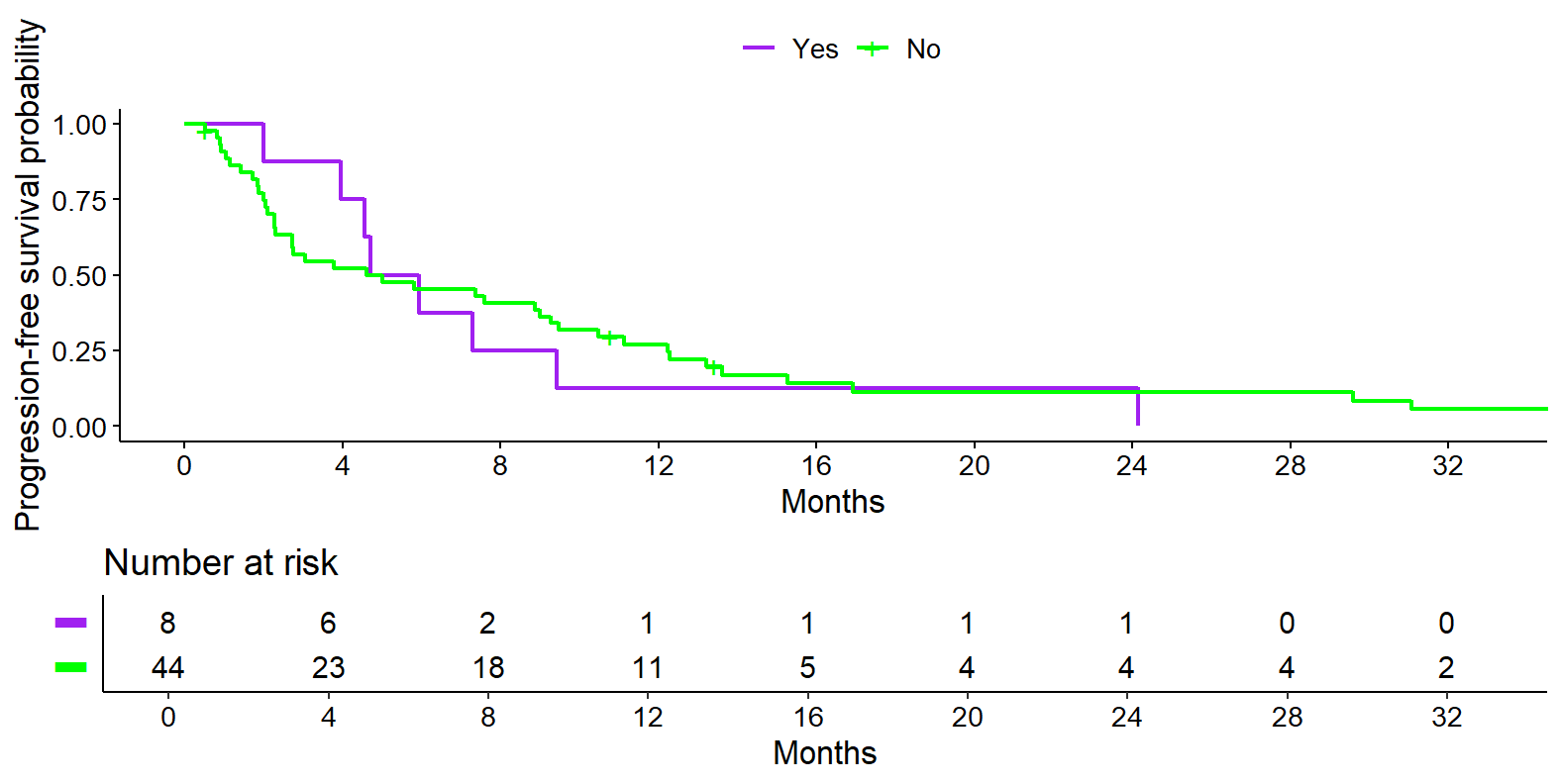


**Figure 5i**: Progression-free survival relative to PIK3CA mutation prognosis


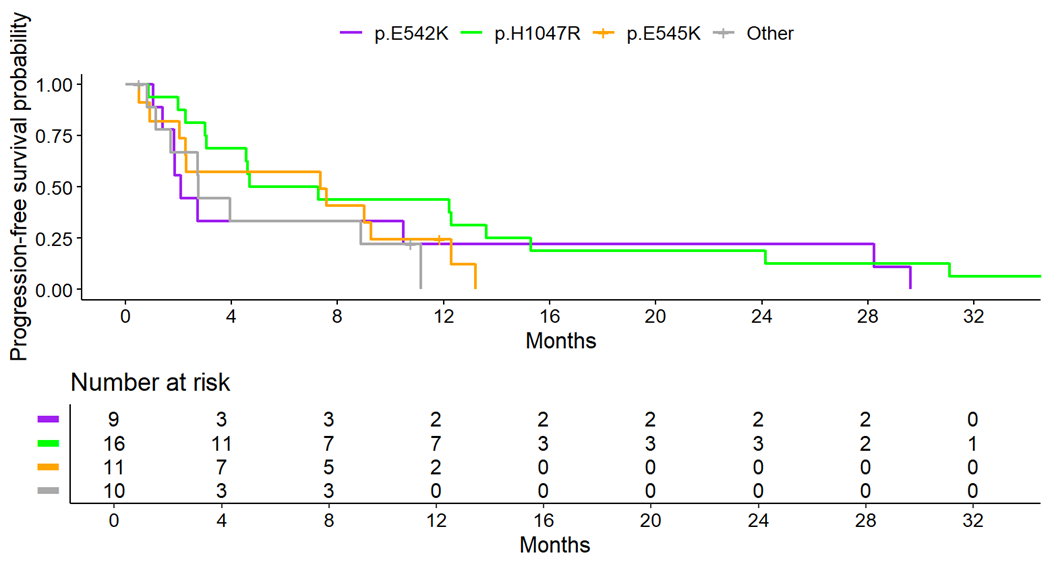


**Figure 5j**: Progression-free survival relative to duration of first line CDK4/6 therapy in

patients receiving second line Alpelisib (<24 months, ≥24 months)


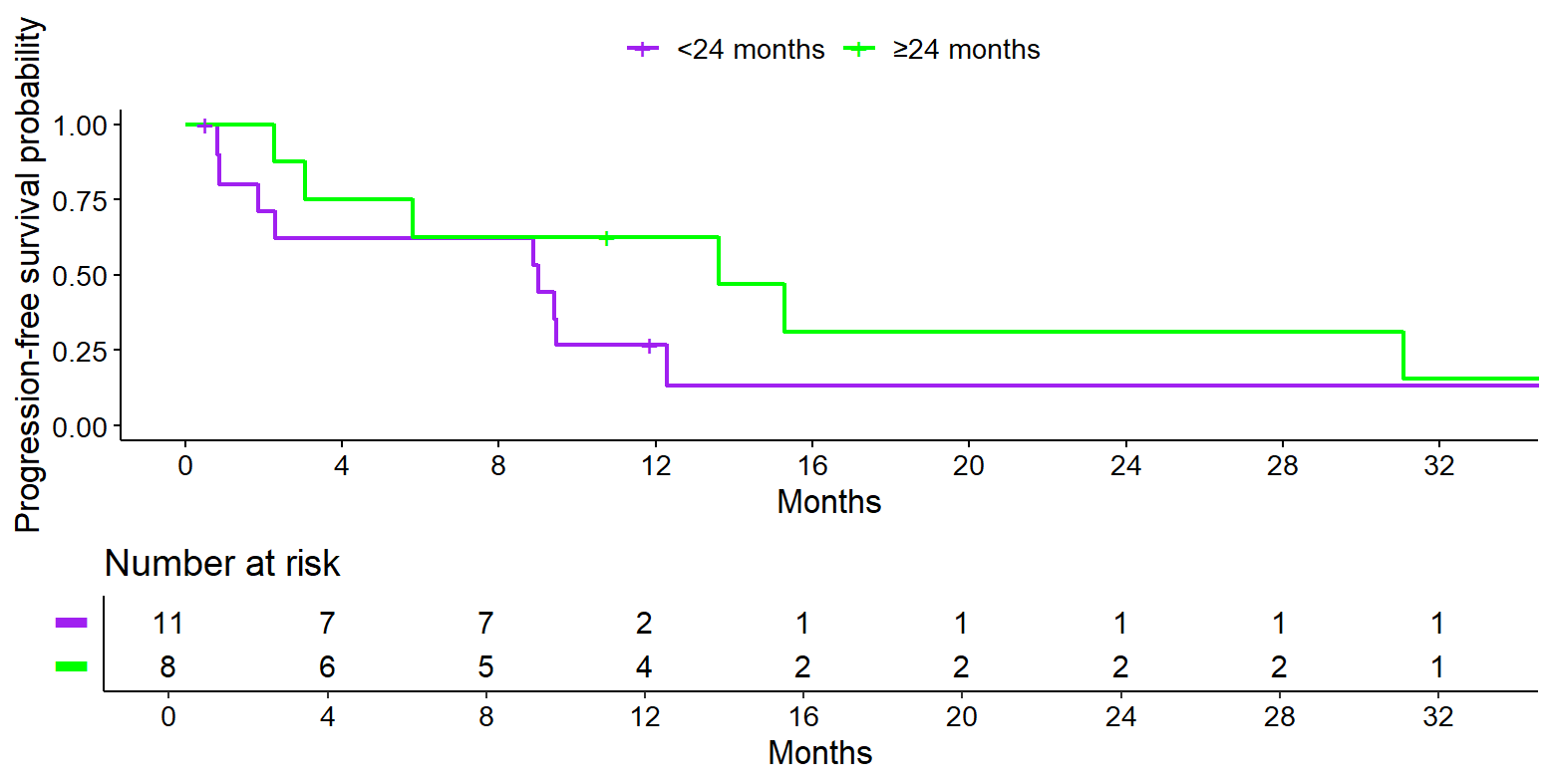


**Figure 6** OS relative to different parameters (age, body mass index, ECOG, tumor grading, metastasis pattern, metastasis timing, number of concomitant diseases, preexisting diabetes, PIK3CA mutation and duration of first-line CDK4/6 therapy)

**Figure 6a**: Overall survival relative to the age (years)


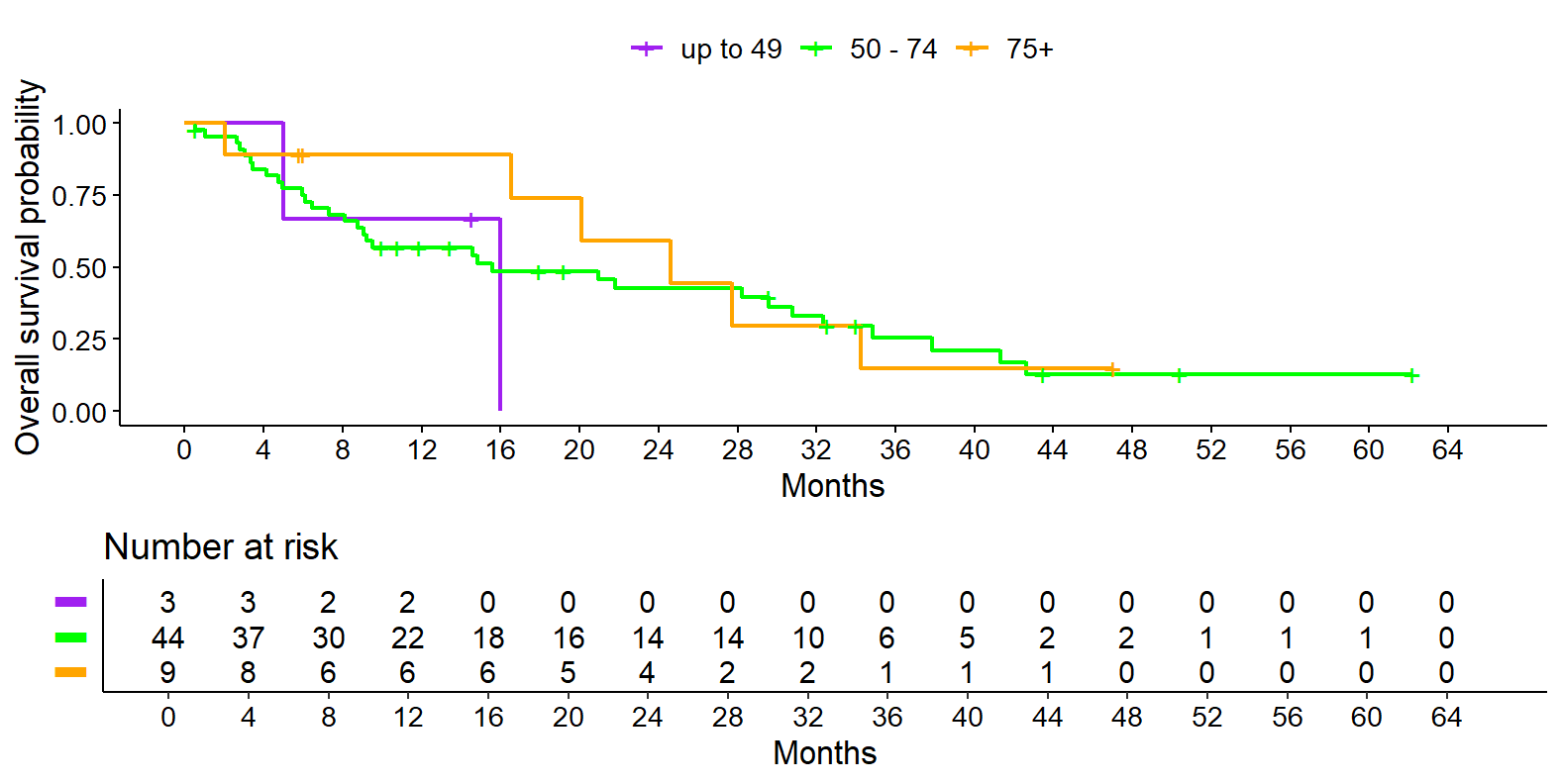


**Figure 6b**: Overall survival relative to the body mass index


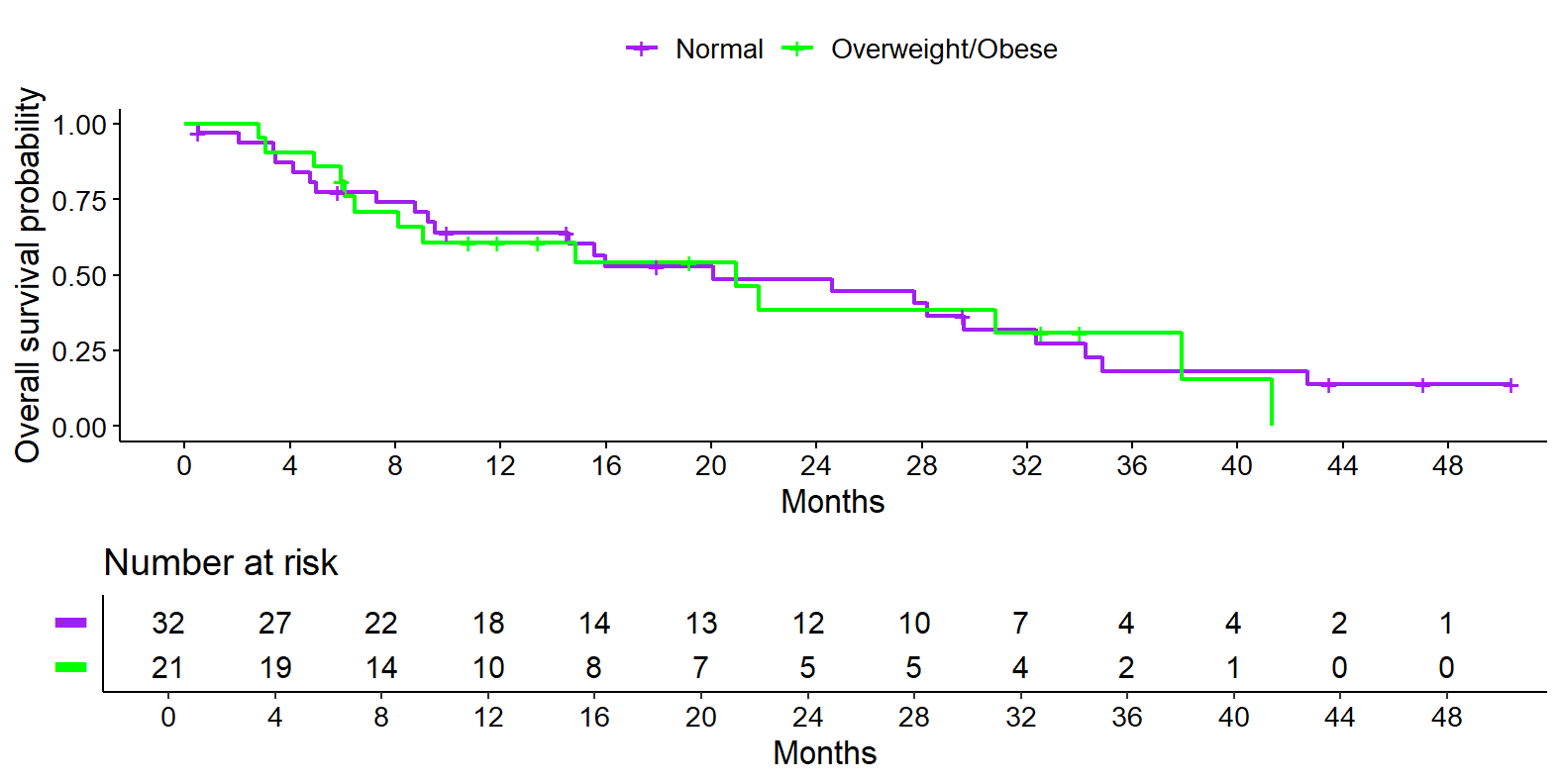


**Figure 6c**: Overall survival relative to the ECOG


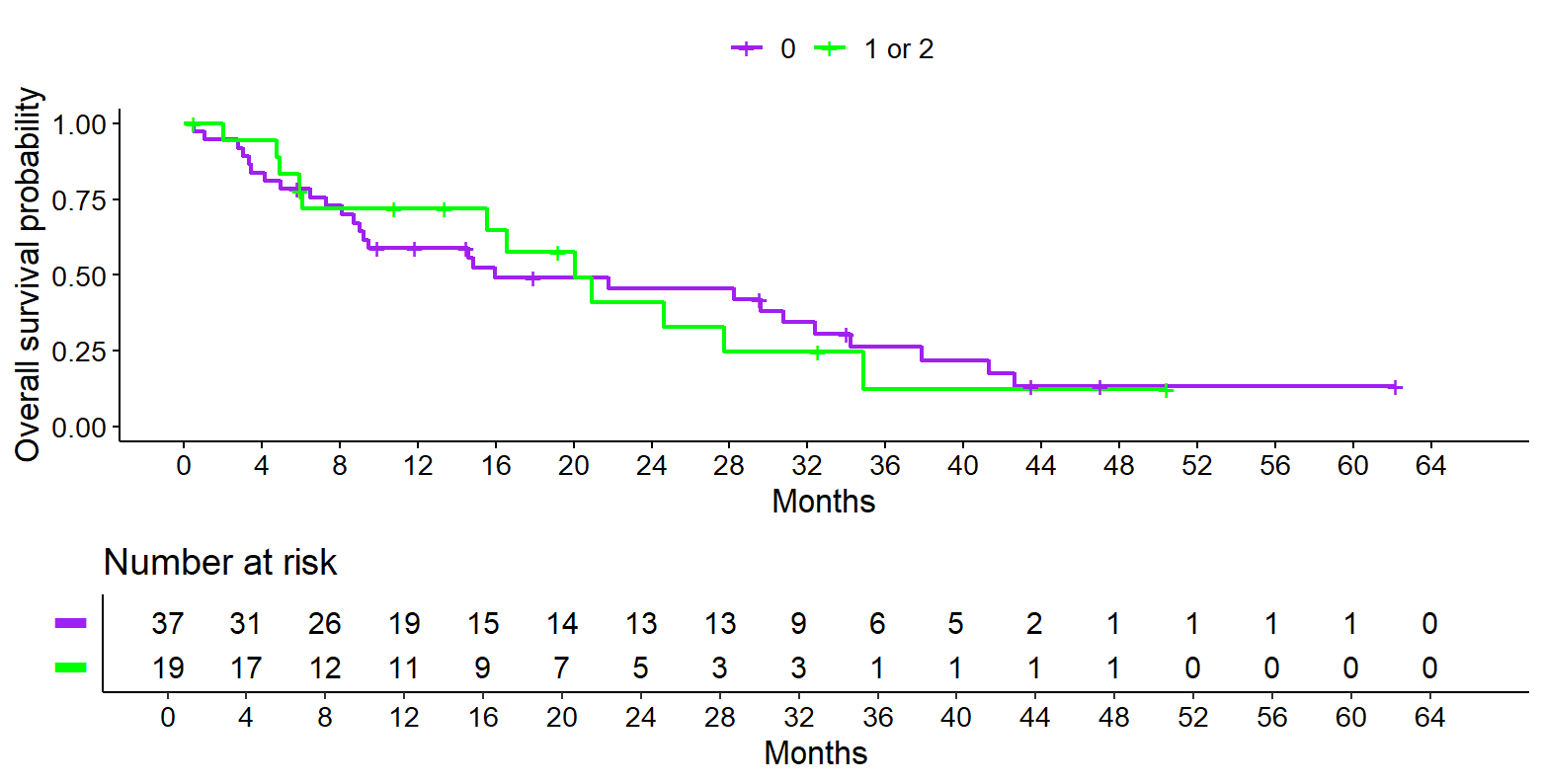


**Figure 6d**: Overall survival relative to the tumor grading


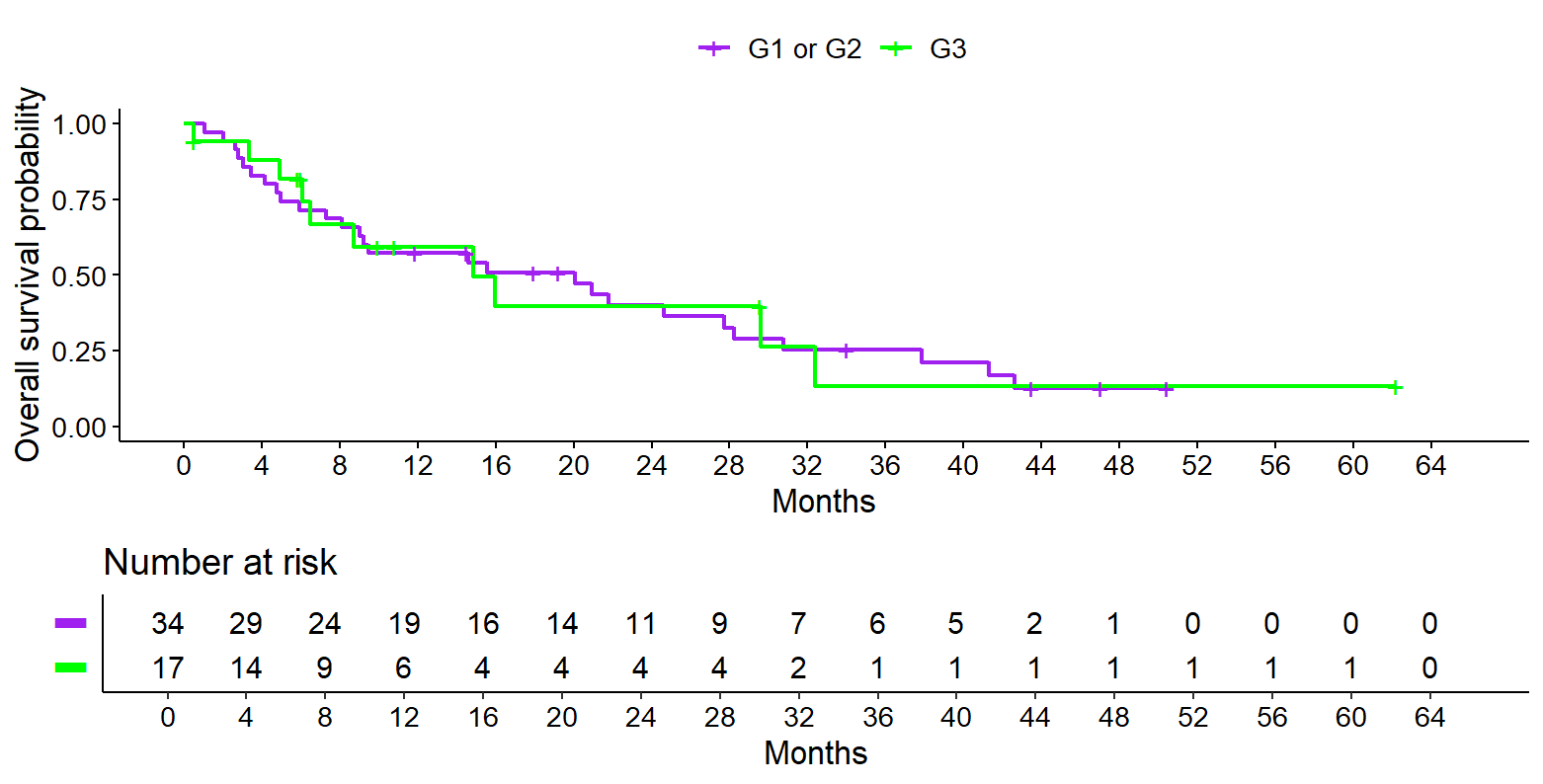


**Figure 6e**: Overall survival relative to metastasis pattern


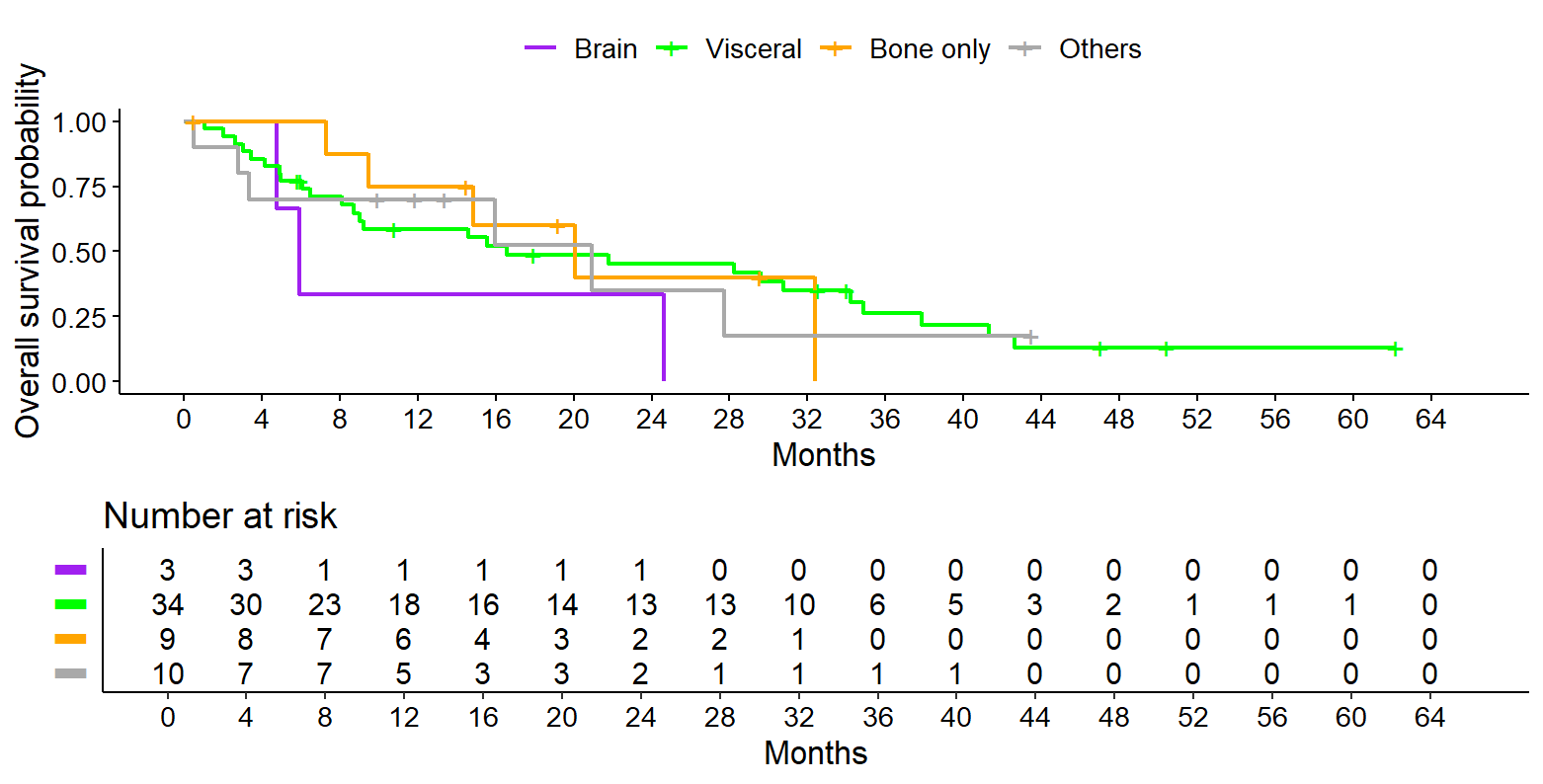


**Figure 6f**: Overall survival relative to metastasis timing (de novo, ≤ 60 months after primary diagnosis, > 60 months after primary diagnosis)


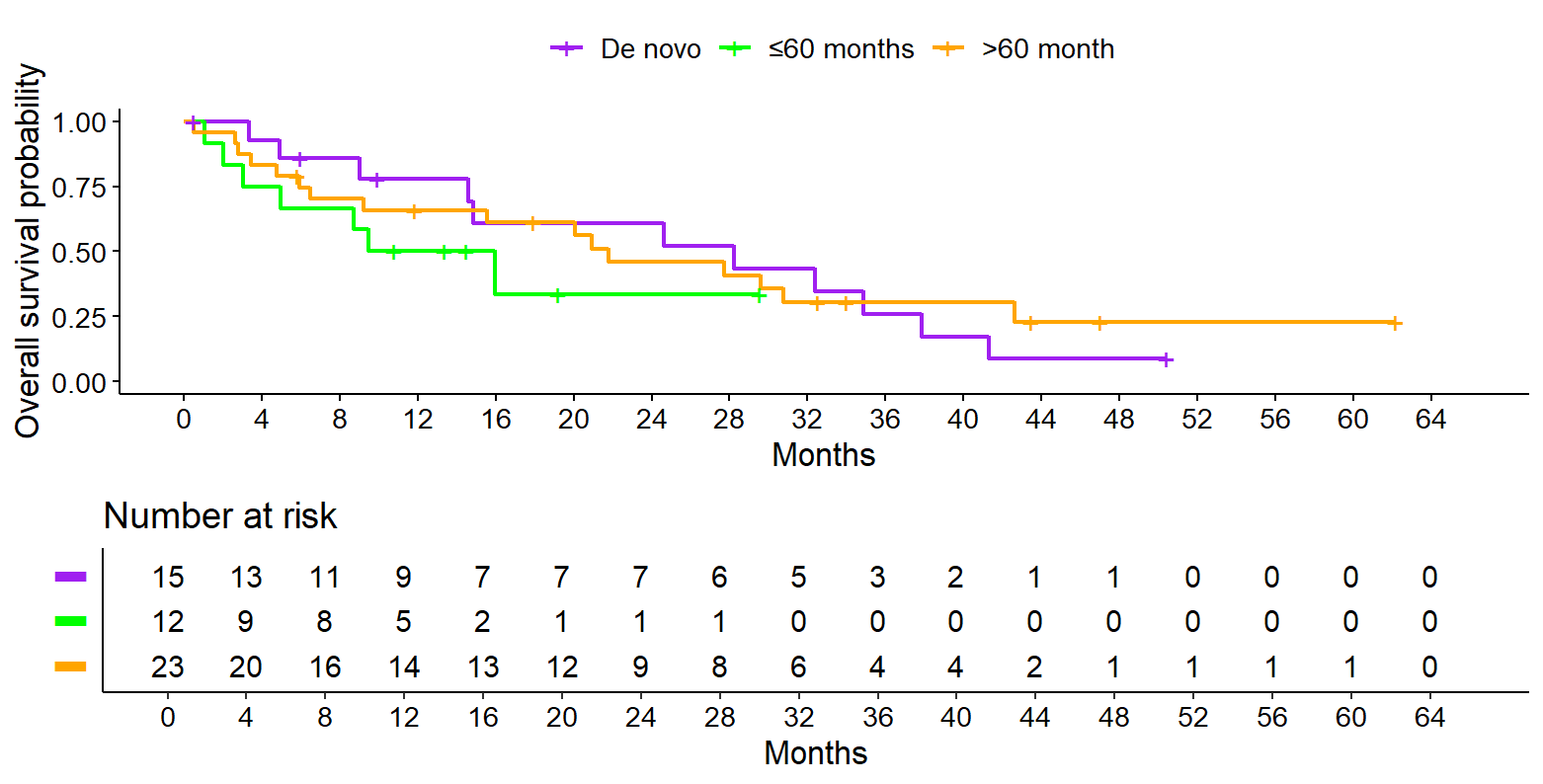


**Figure 6g**: Overall survival relative to the number of concomitant diseases


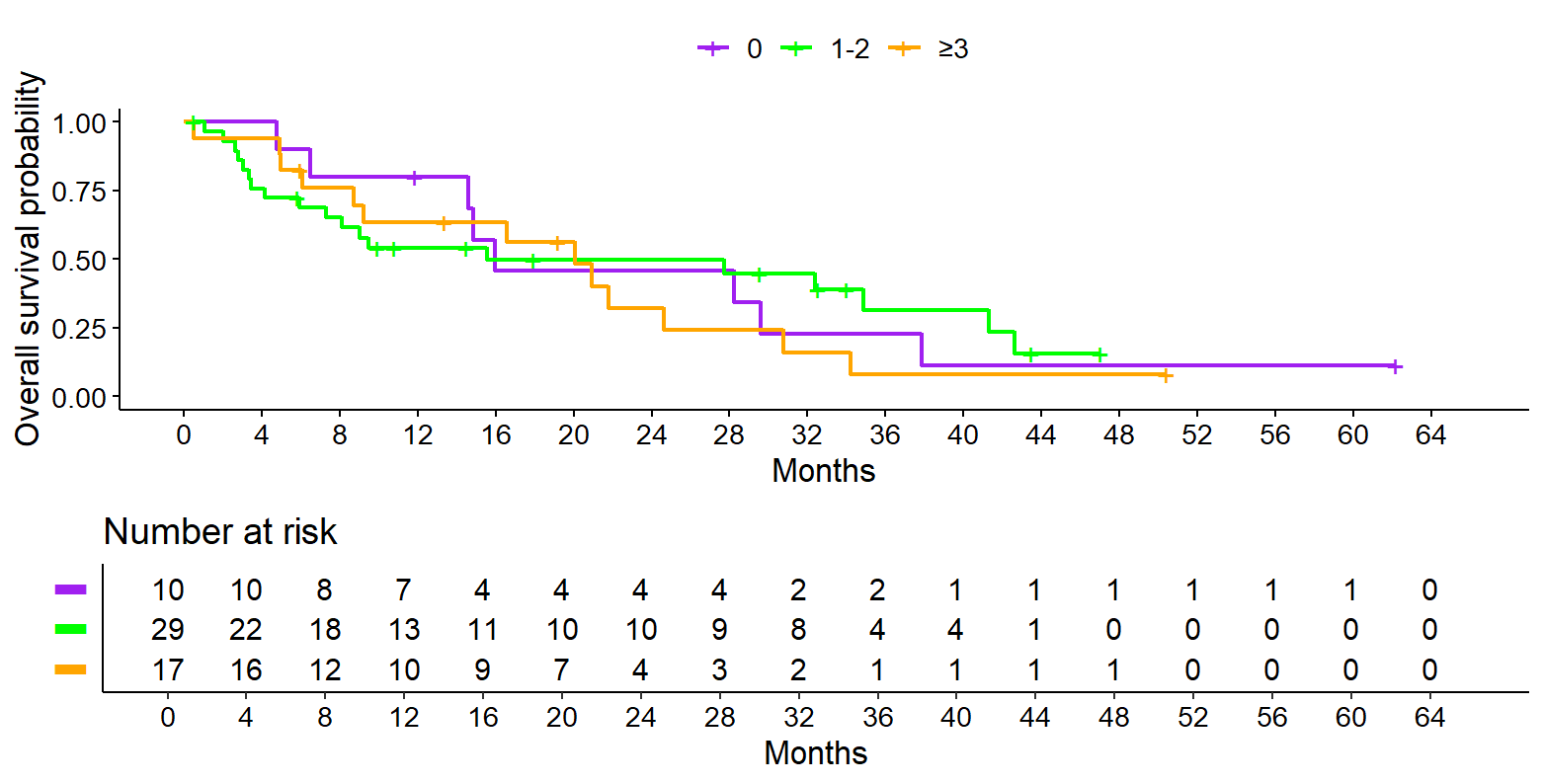


**Figure 6h**: Overall survival relative to diabetes (yes/no)


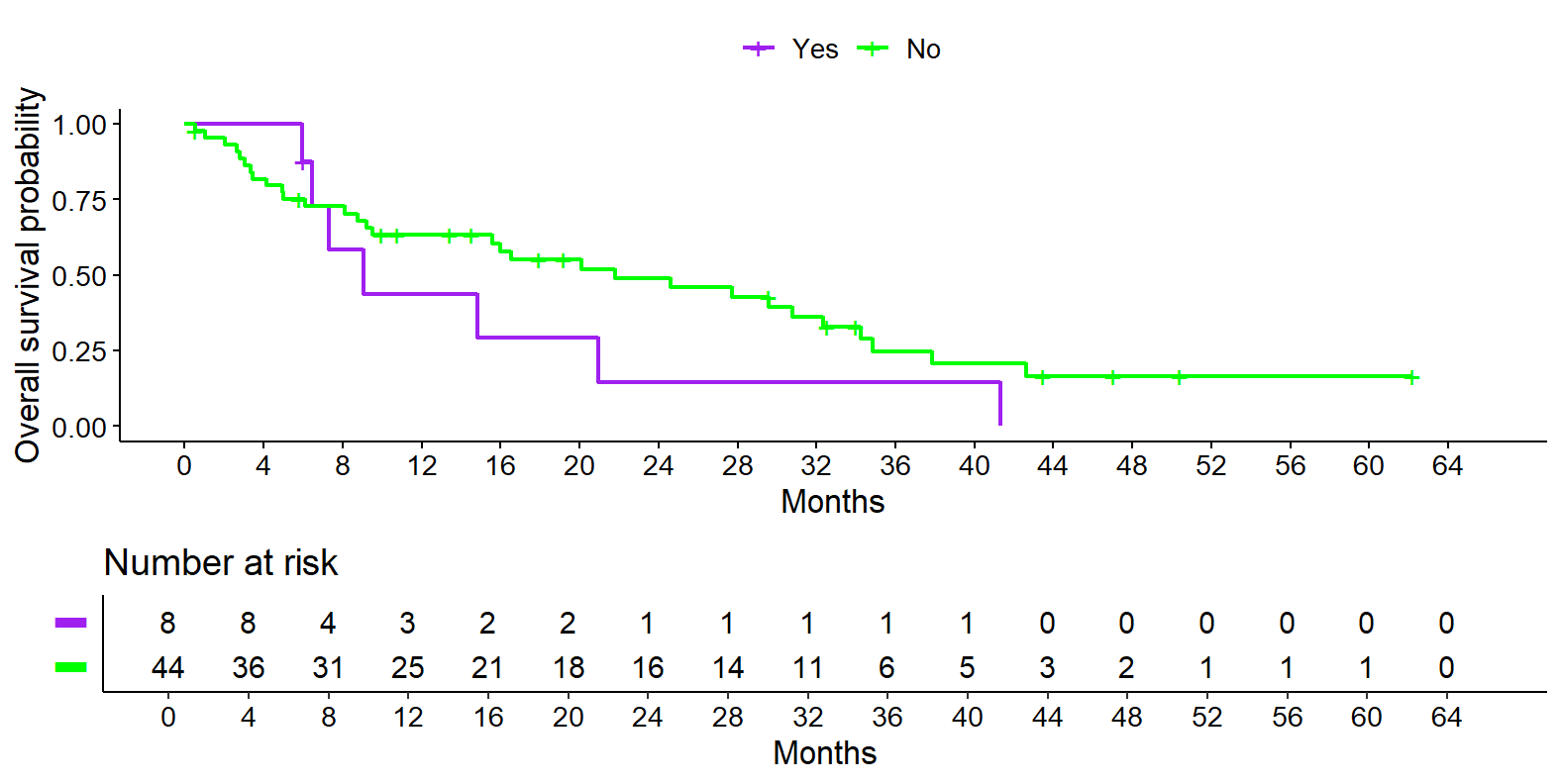


**Figure 6i**: Overall survival relative to PIK3CA mutation prognosis


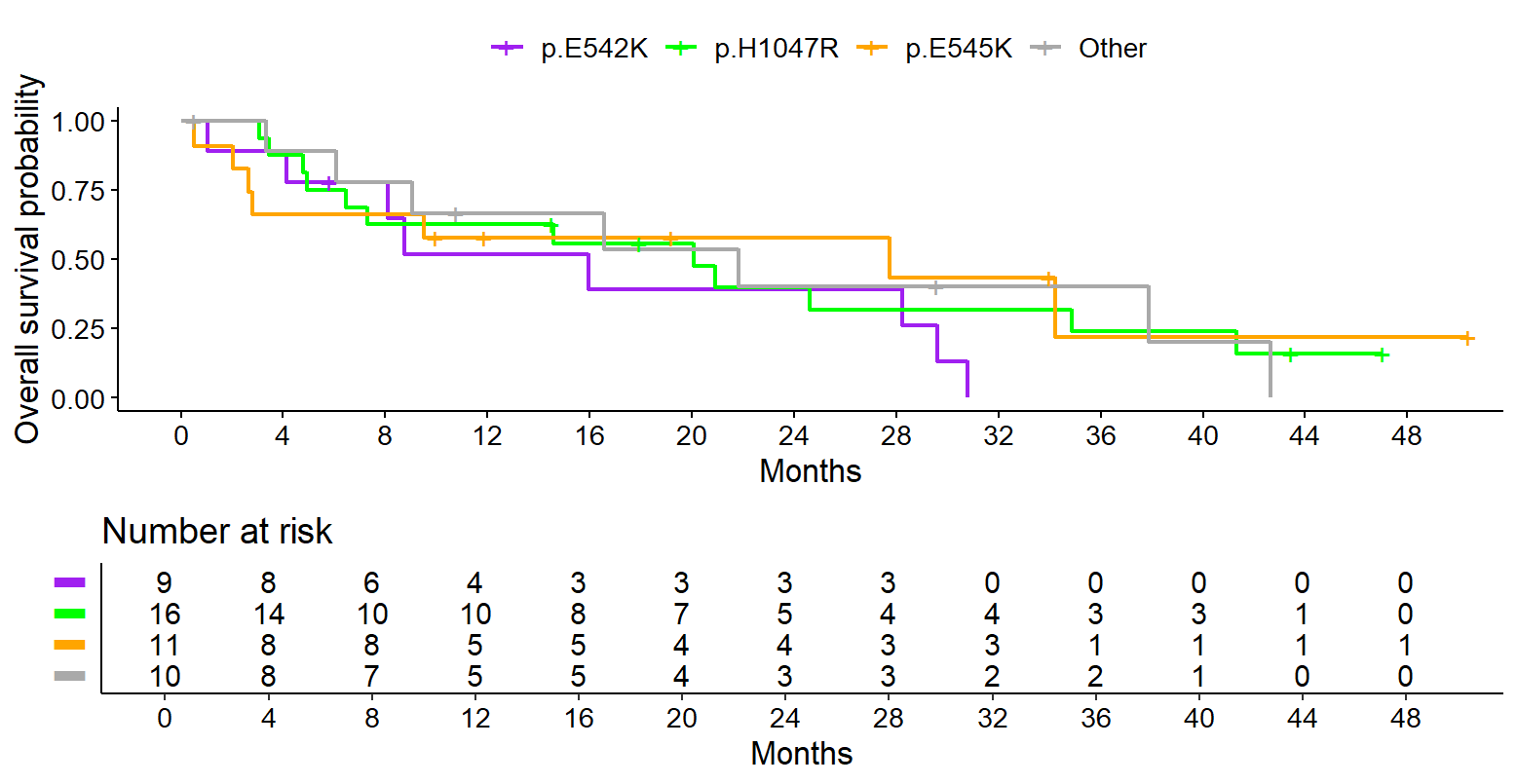


**Figure 6j:** Overall survival relative to duration of first line CDK4/6 therapy in

patients receiving second line Alpelisib (<24 months, ≥24 months)


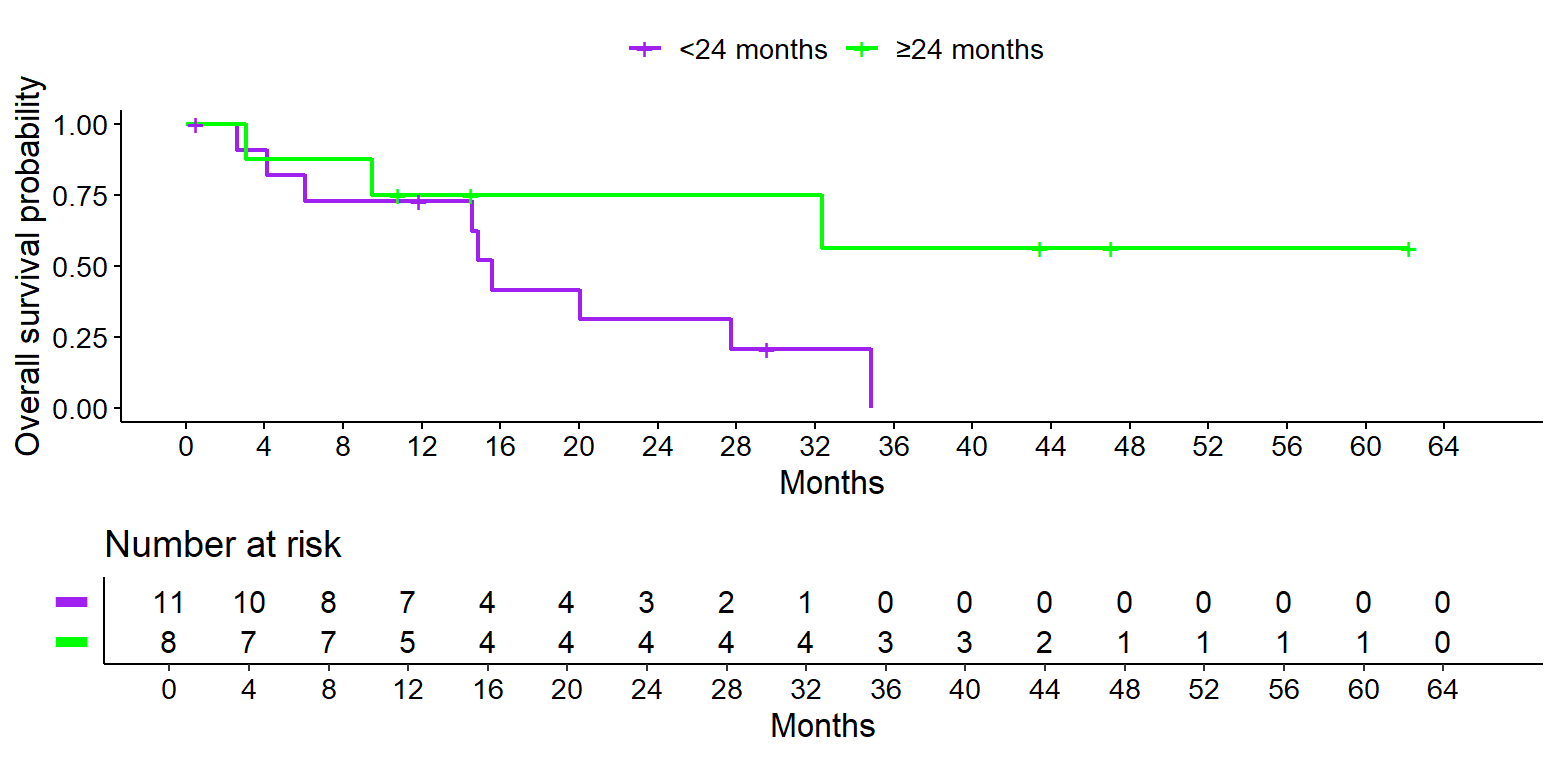

Supplement: Supplementary file 1 — Supplementary file1 (DOCX 472 KB) [file 10549_2026_7939_MOESM1_ESM.docx]
